# Supplementary figures and images for: Glucosylceramide synthase regulates hepatocyte repair after concanavalin A-induced immune-mediated liver injury
Source: PeerJ. 2021 Sep 14;9:e12138. doi: 10.7717/peerj.12138 (PMC8447939; doi:10.7717/peerj.12138)

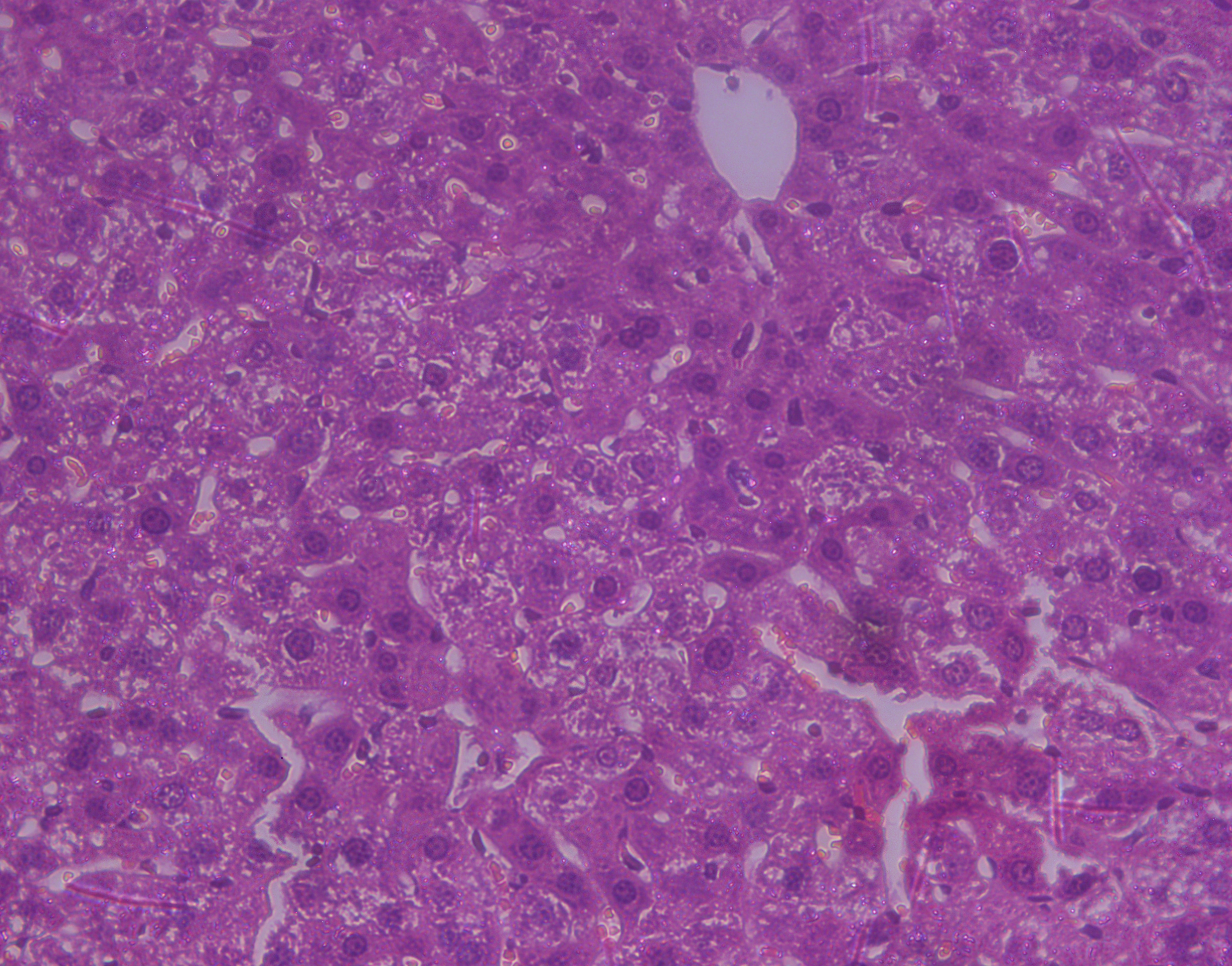

Supplement: Supplemental Information 1 [file peerj-09-12138-s001.zip › Fig 2A, 2F/40x.tif]

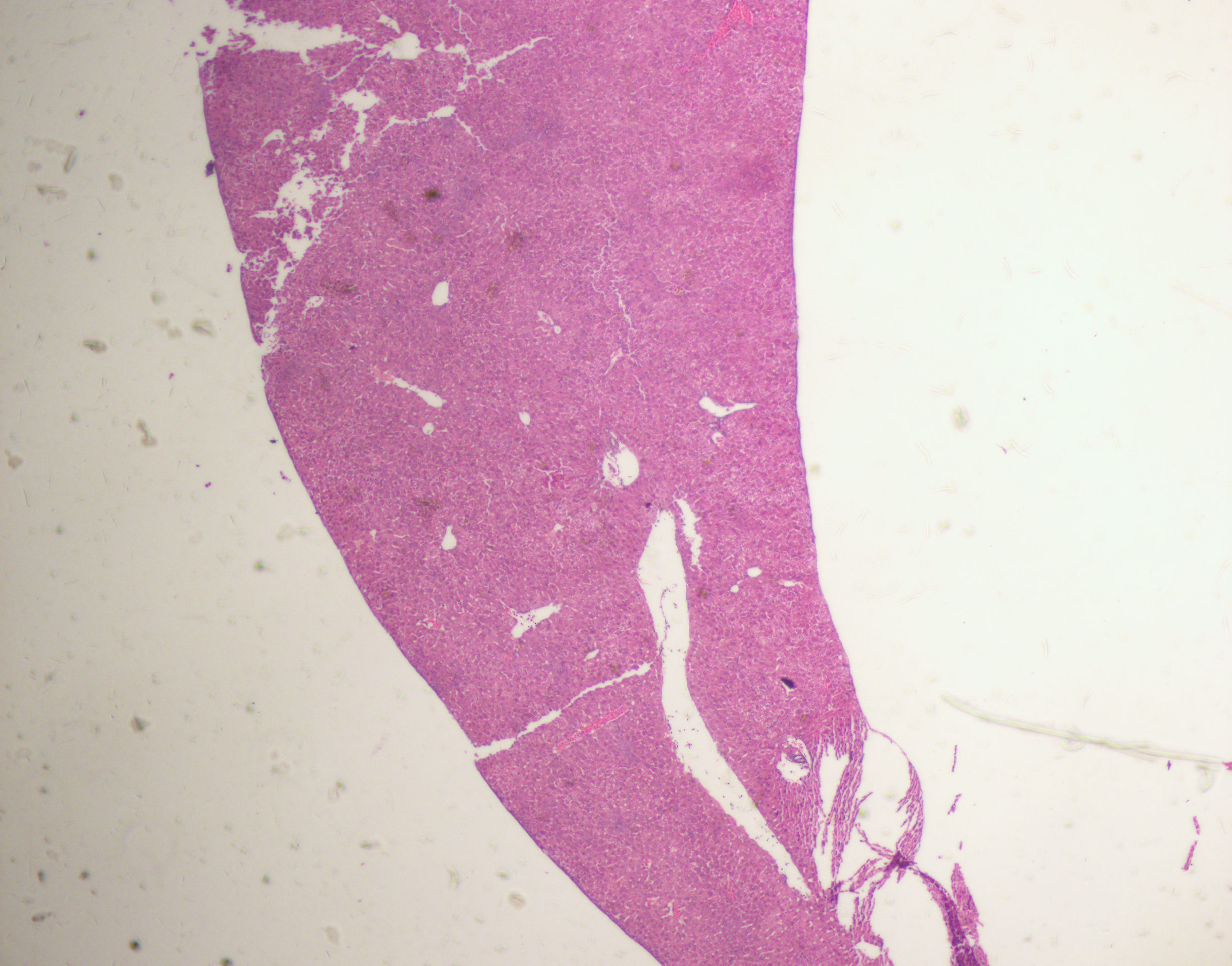

Supplement: Supplemental Information 1 [file peerj-09-12138-s001.zip › Fig 2A, 2F/4x.tif]

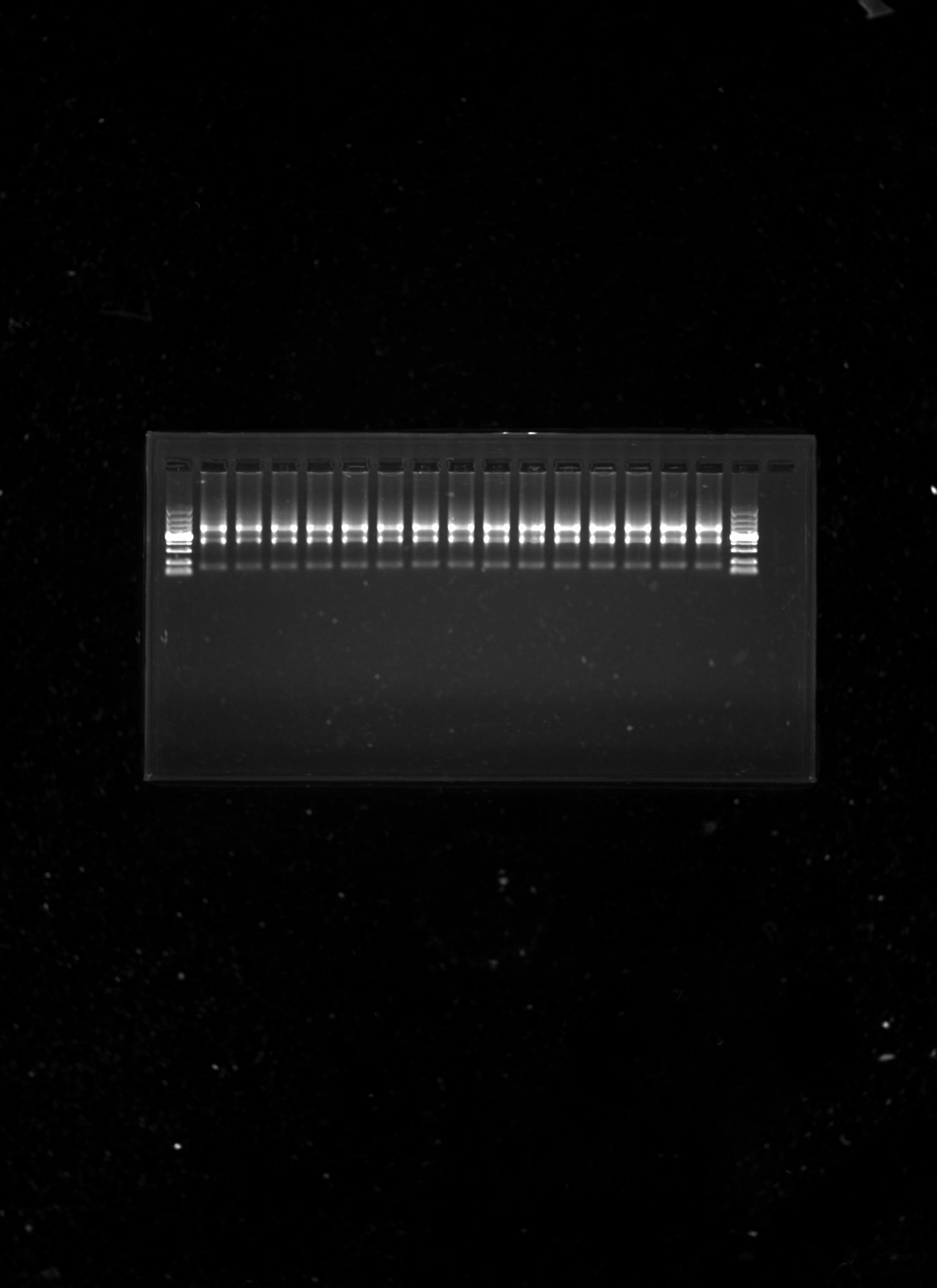

Supplement: Supplemental Information 1 [file peerj-09-12138-s001.zip › Fig 1D/RNA.jpg]

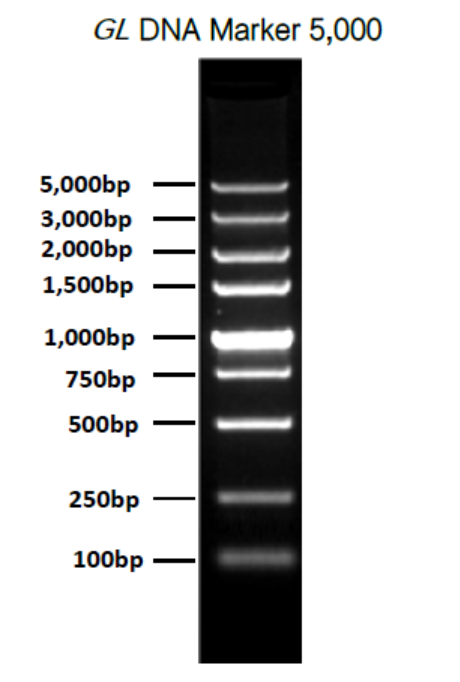

Supplement: Supplemental Information 1 [file peerj-09-12138-s001.zip › Fig 1D/搜狗截图20210704221811.png]

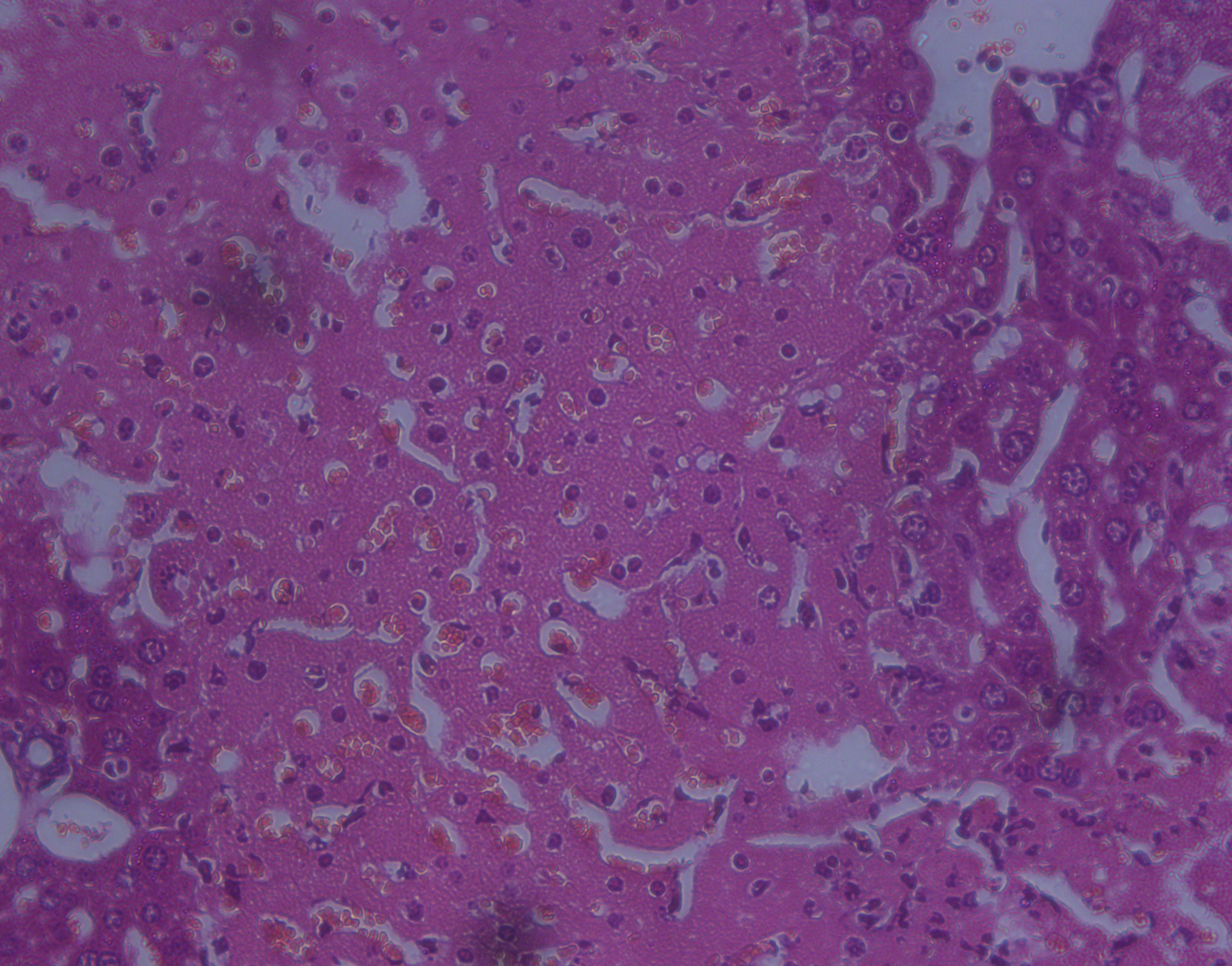

Supplement: Supplemental Information 2 [file peerj-09-12138-s002.zip › Fig 2B, 2G/40x.tif]

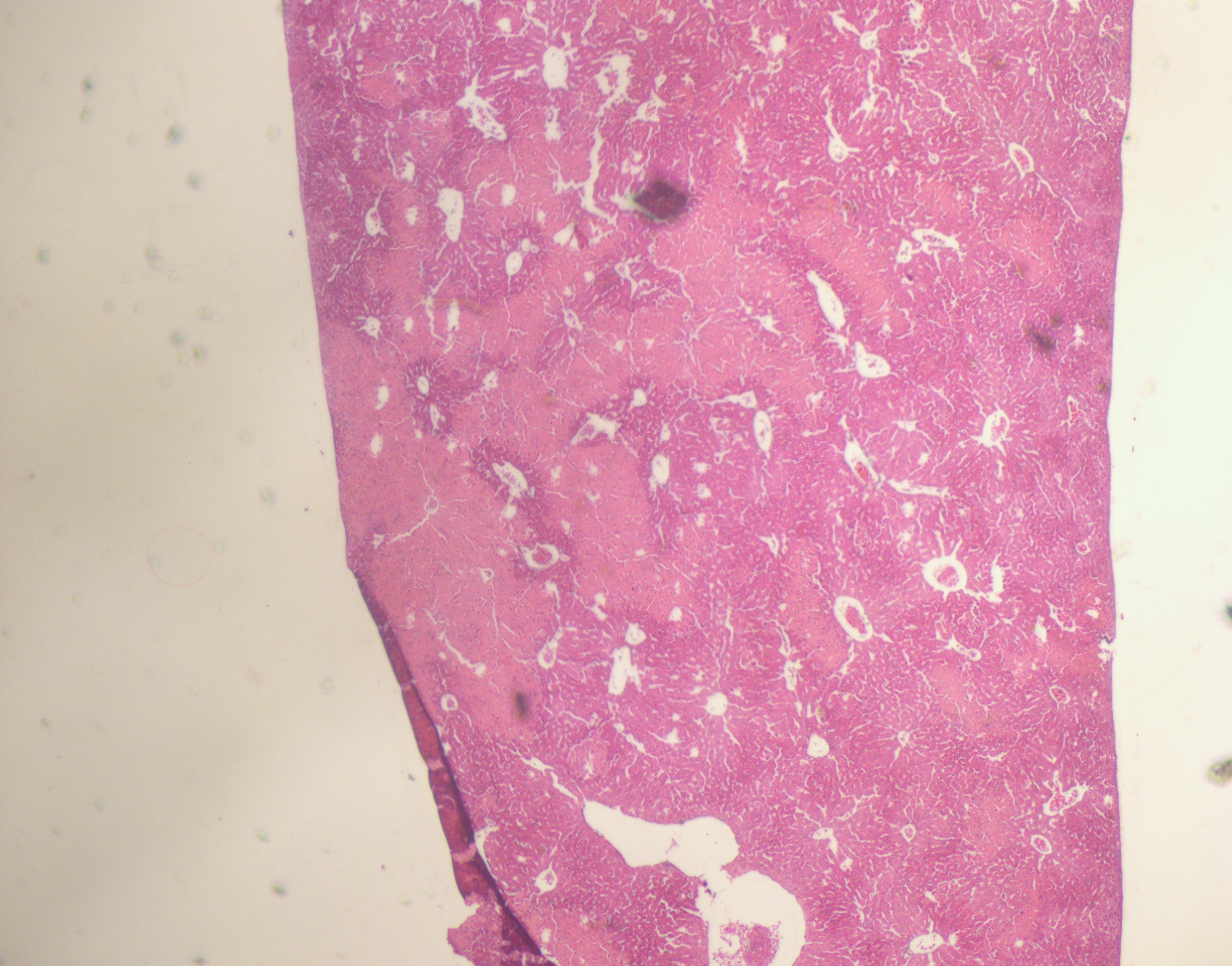

Supplement: Supplemental Information 2 [file peerj-09-12138-s002.zip › Fig 2B, 2G/4x.tif]

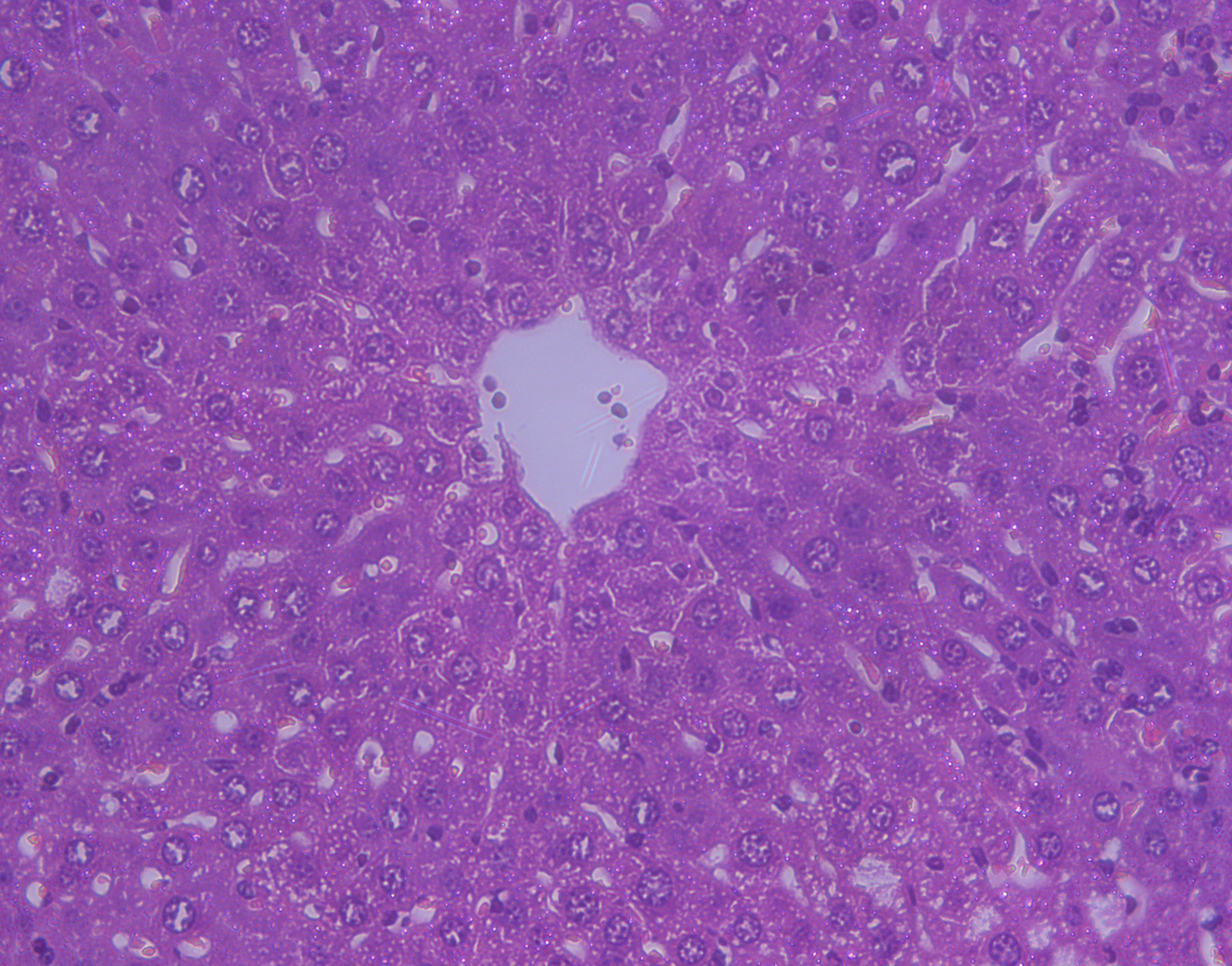

Supplement: Supplemental Information 3 [file peerj-09-12138-s003.zip › Fig 2C, 2H/40x.tif]

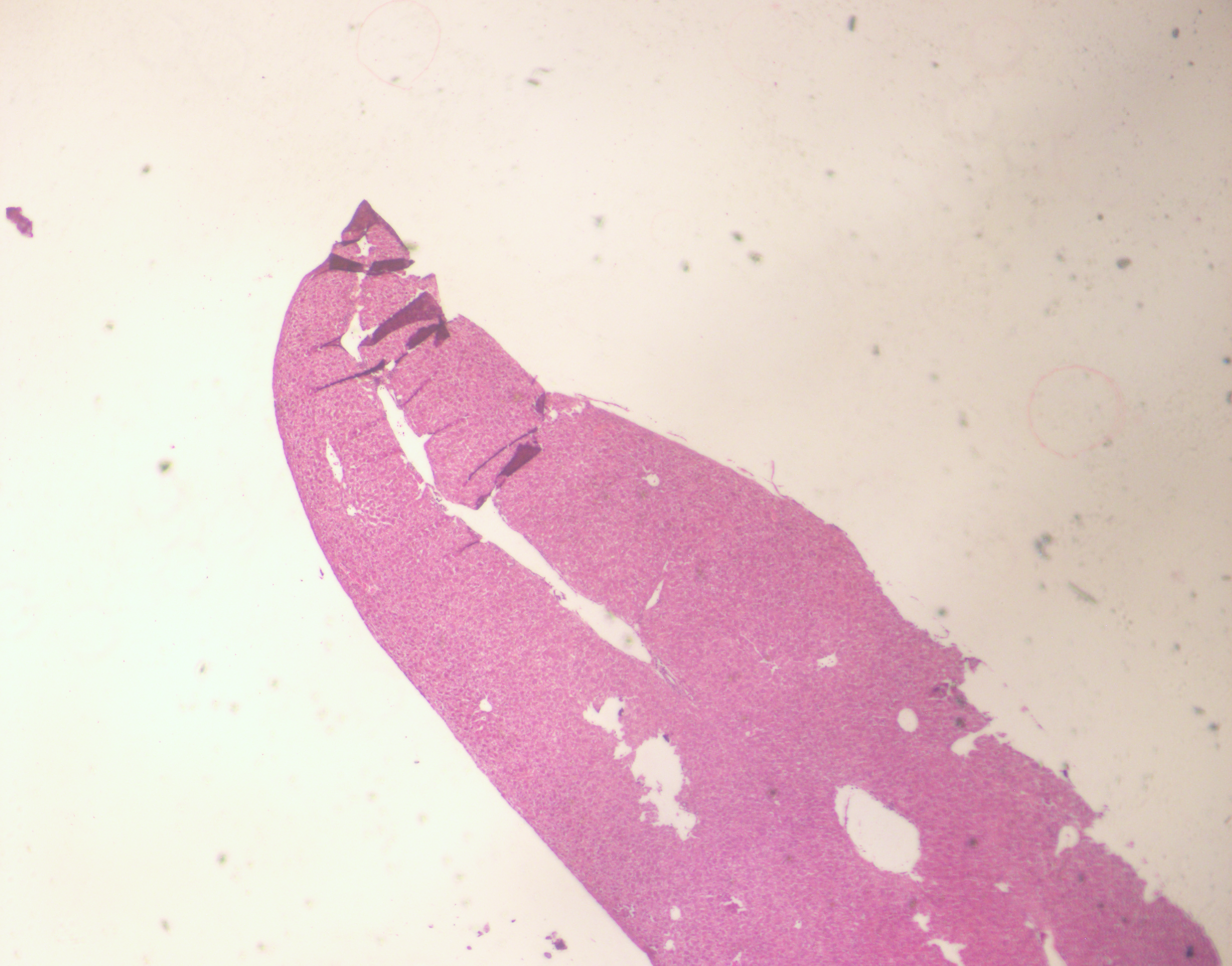

Supplement: Supplemental Information 3 [file peerj-09-12138-s003.zip › Fig 2C, 2H/4x.tif]

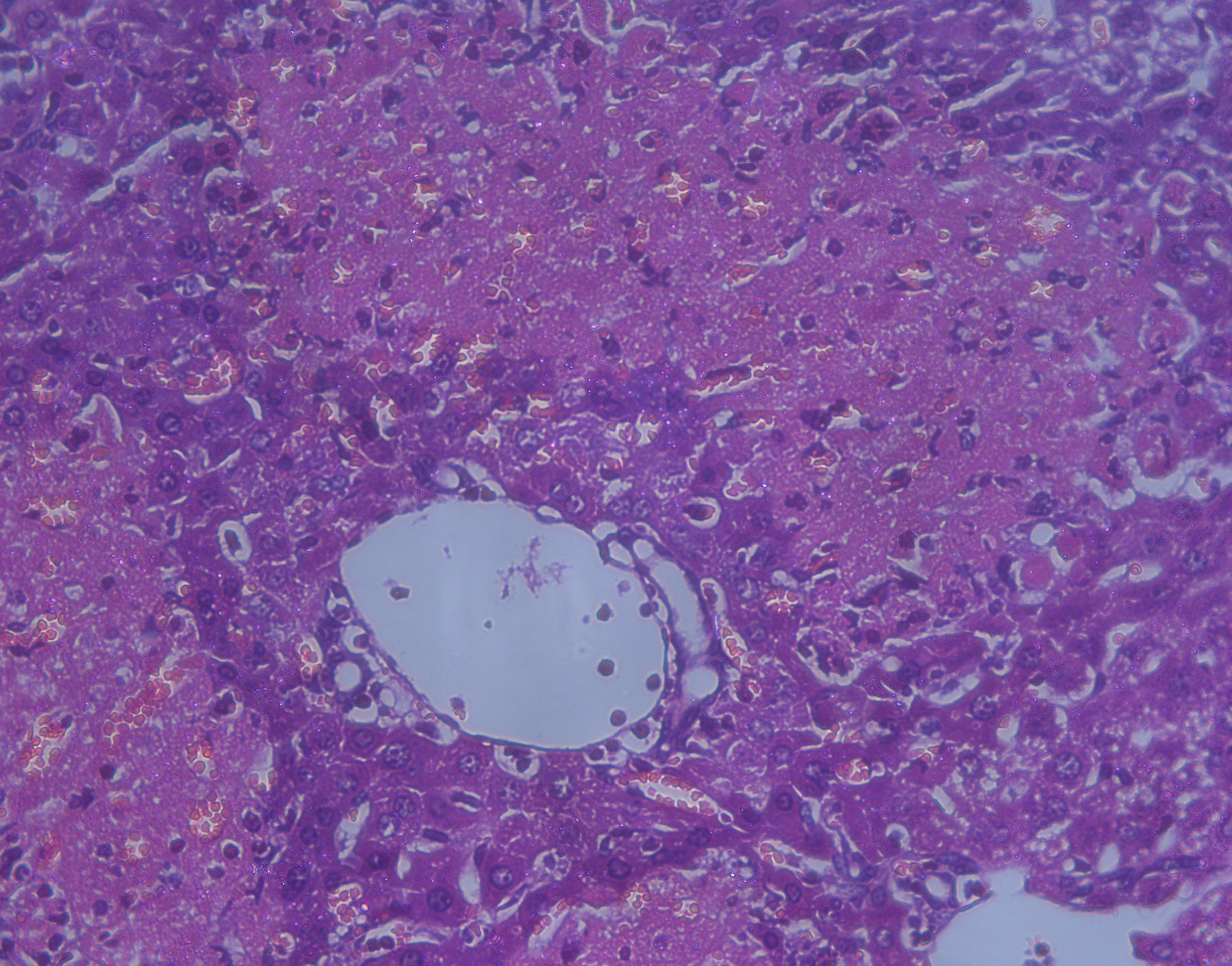

Supplement: Supplemental Information 4 [file peerj-09-12138-s004.zip › Fig 2D, 2I/40x.tif]

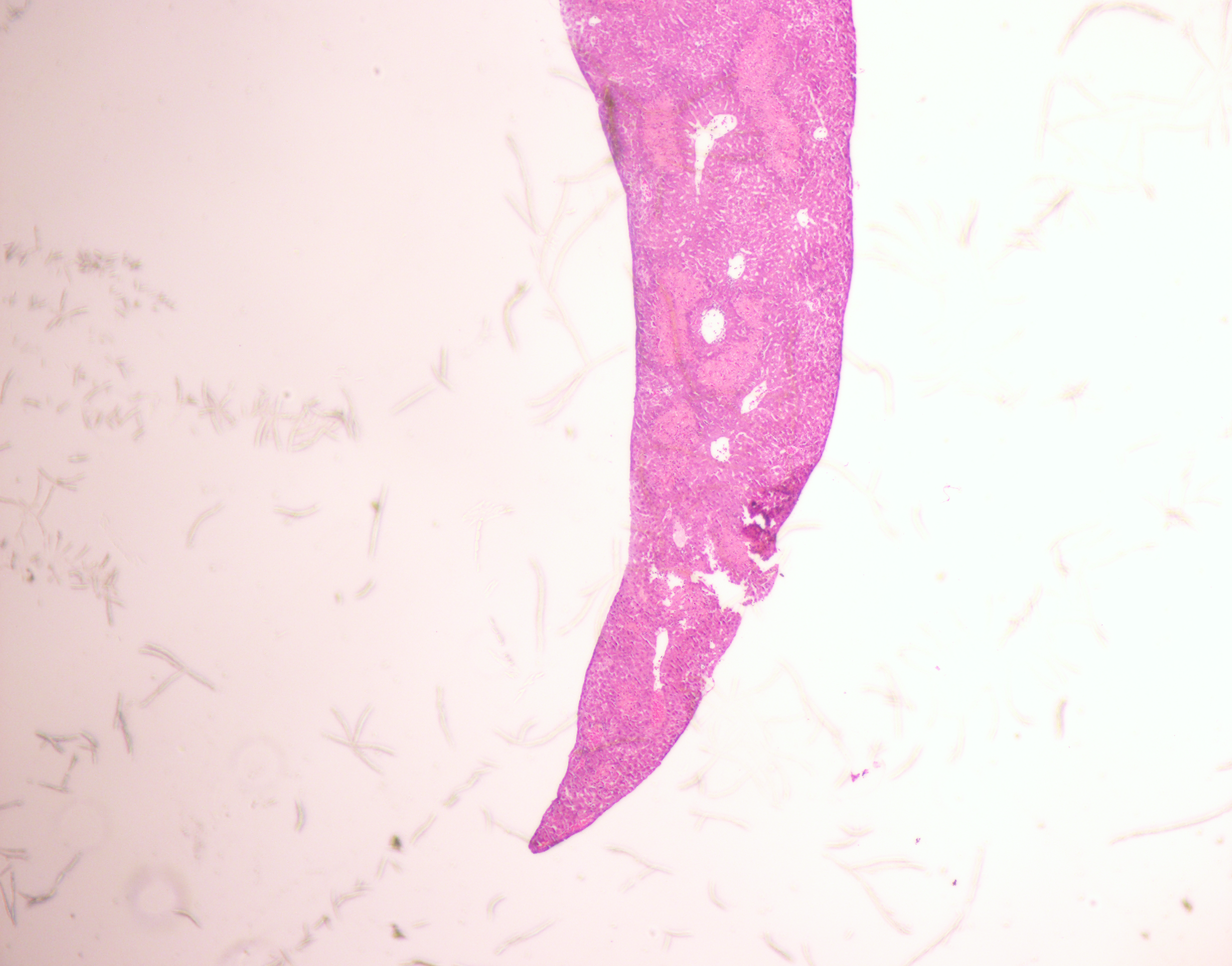

Supplement: Supplemental Information 4 [file peerj-09-12138-s004.zip › Fig 2D, 2I/4x.tif]

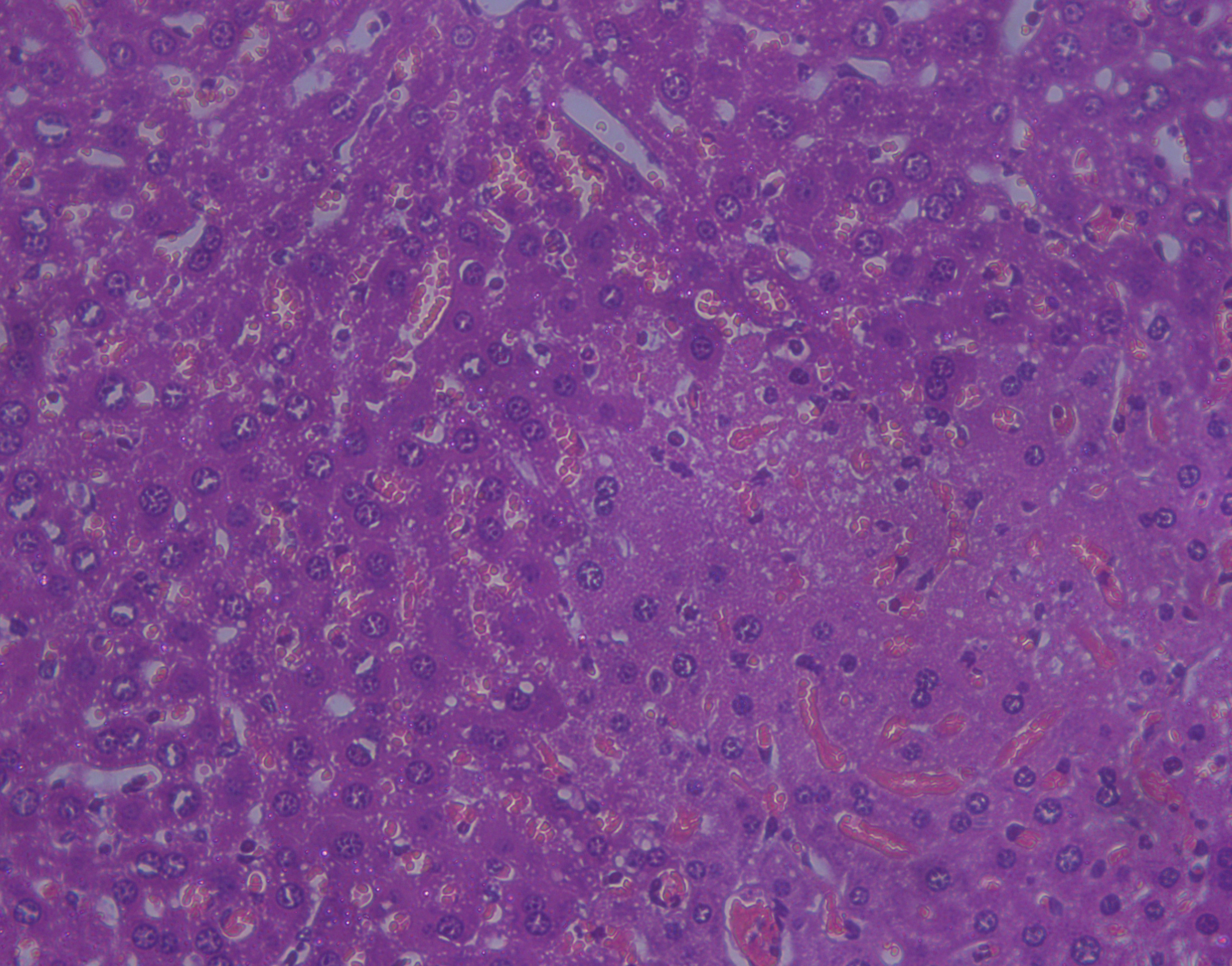

Supplement: Supplemental Information 5 [file peerj-09-12138-s005.zip › Fig 2E, 2J/40x.tif]

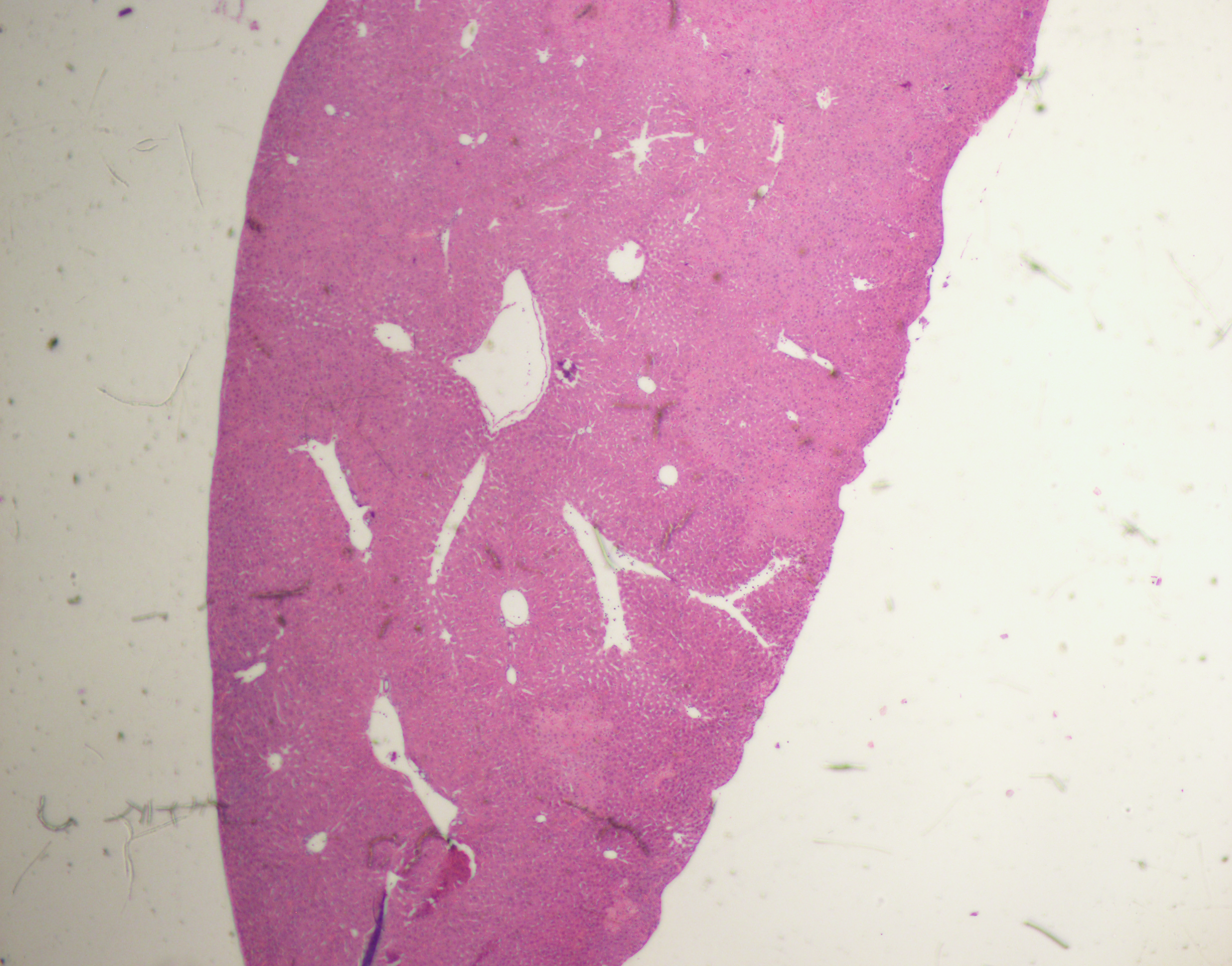

Supplement: Supplemental Information 5 [file peerj-09-12138-s005.zip › Fig 2E, 2J/4x.tif]

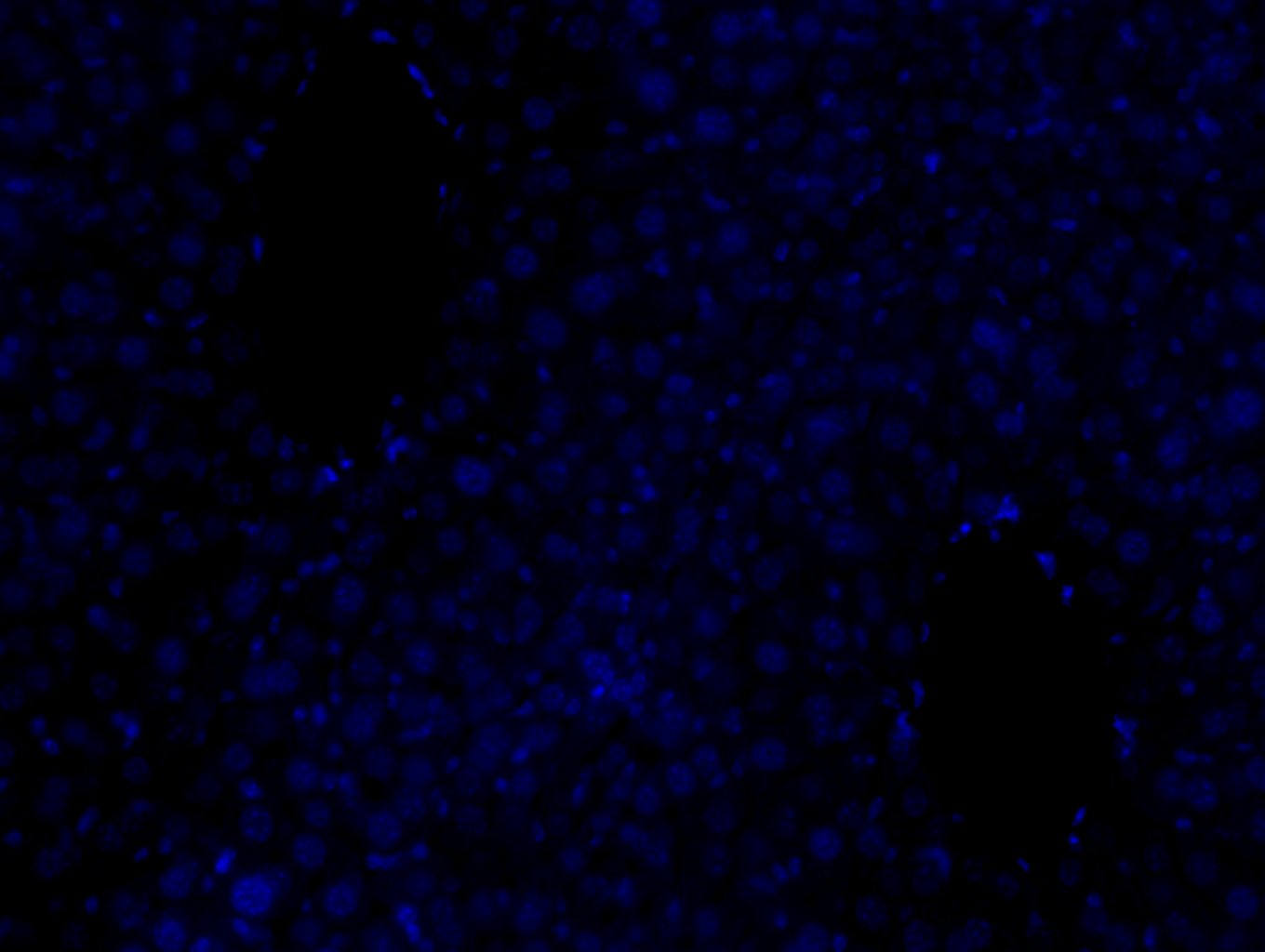

Supplement: Supplemental Information 5 [file peerj-09-12138-s005.zip › Fig 3A/ConA DAPI.jpg]

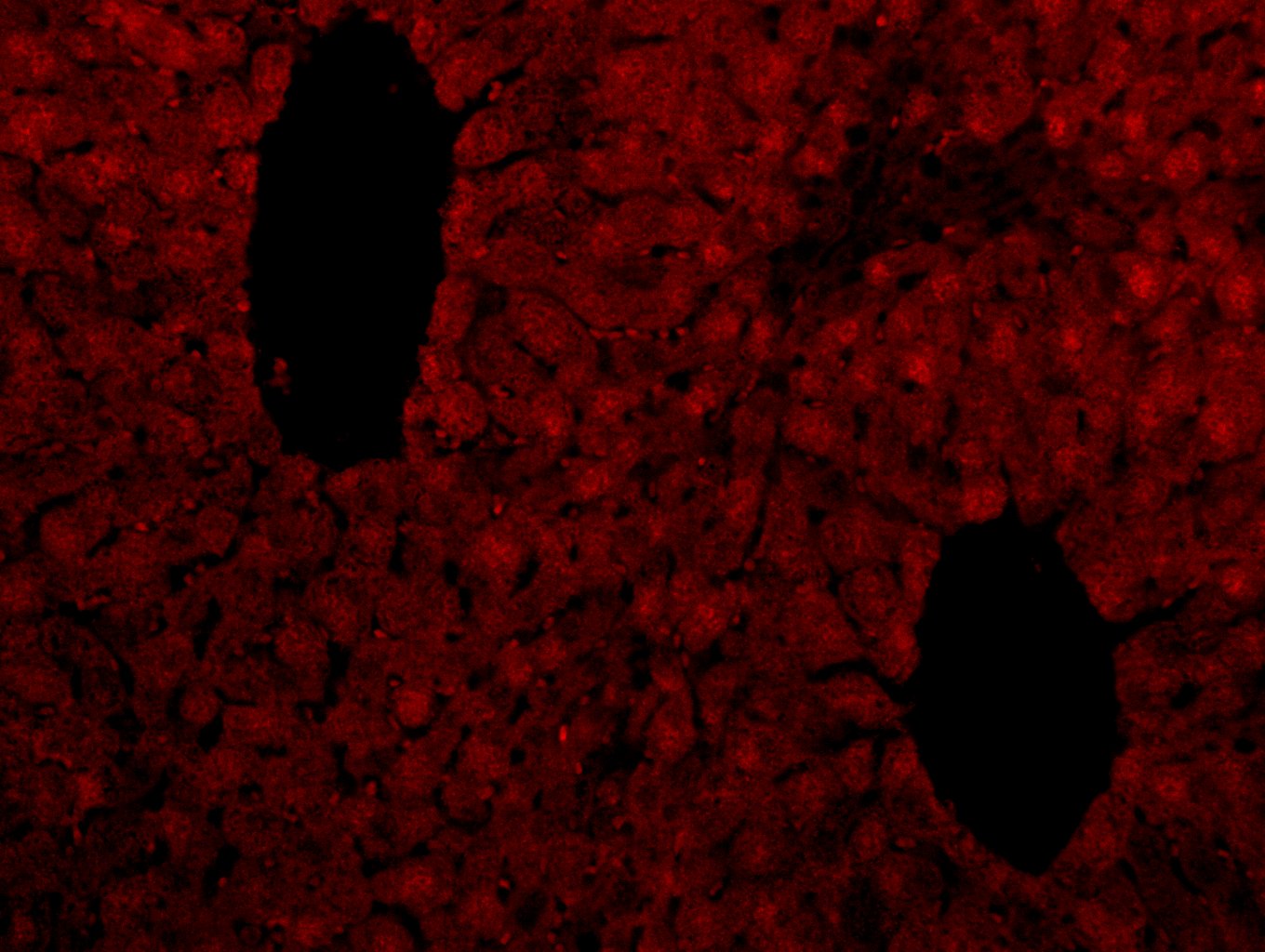

Supplement: Supplemental Information 5 [file peerj-09-12138-s005.zip › Fig 3A/ConA GCS.jpg]

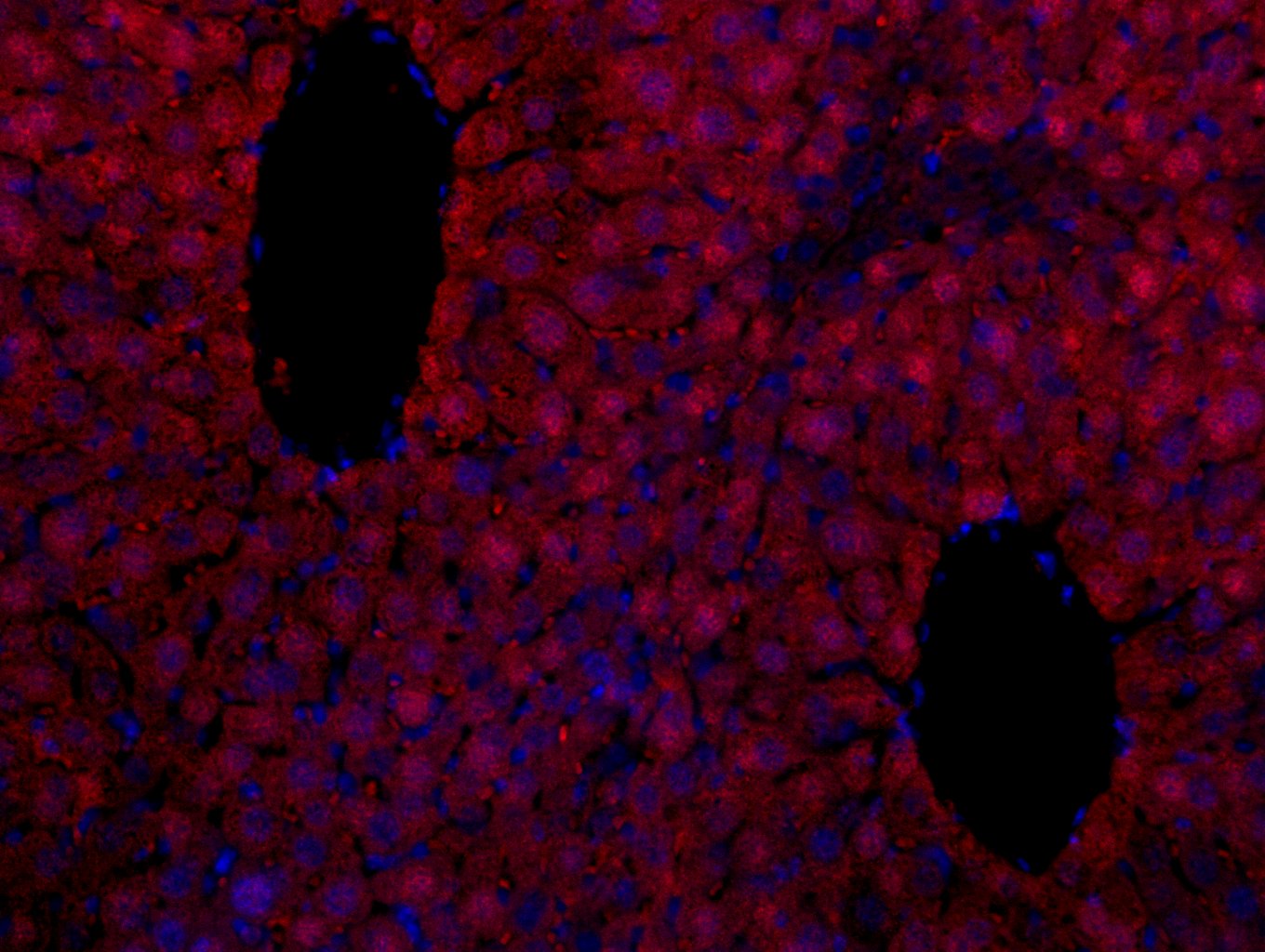

Supplement: Supplemental Information 5 [file peerj-09-12138-s005.zip › Fig 3A/ConA merge.jpg]

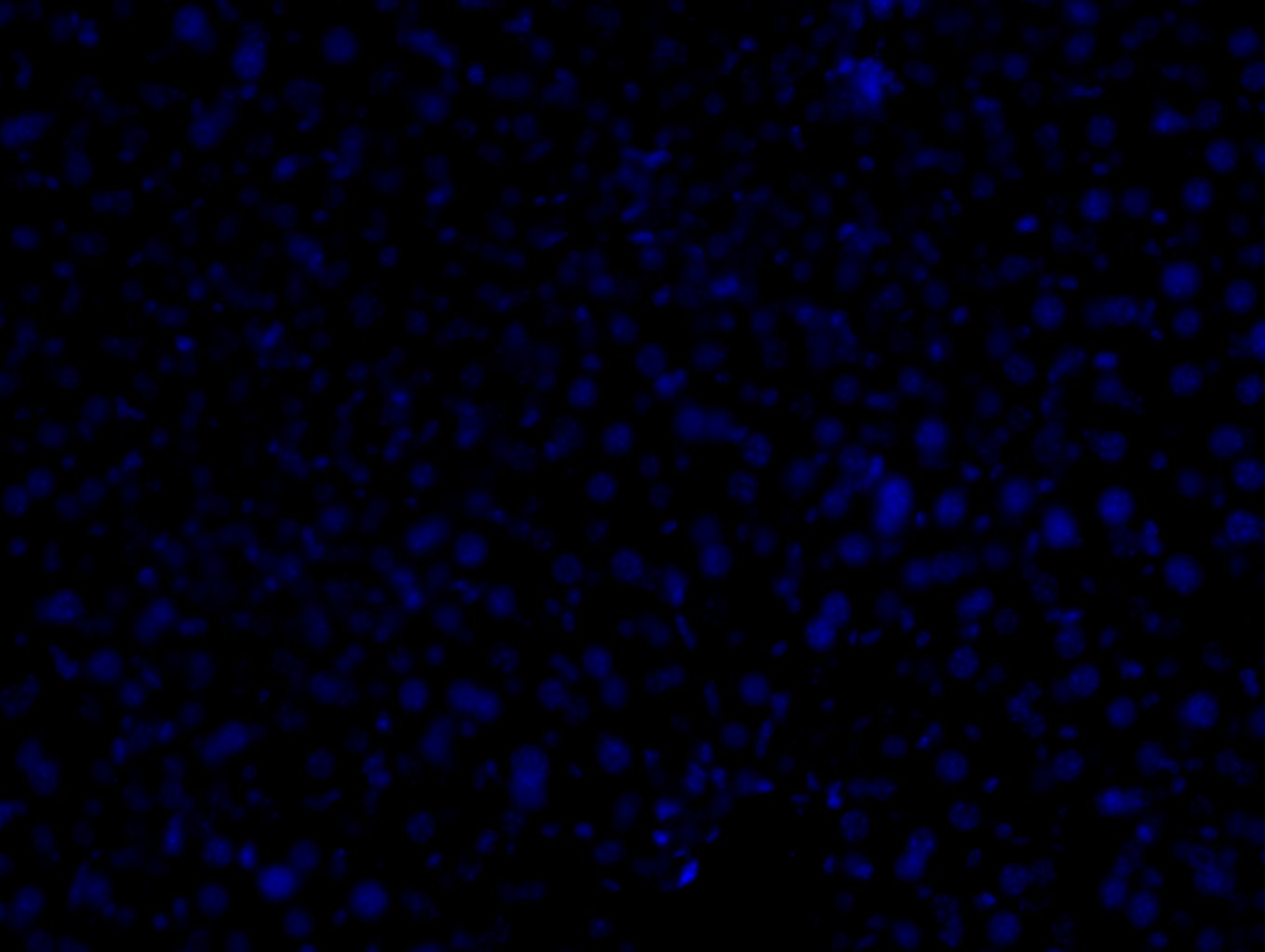

Supplement: Supplemental Information 5 [file peerj-09-12138-s005.zip › Fig 3A/GCS siRNA+ConA DAPI.jpg]

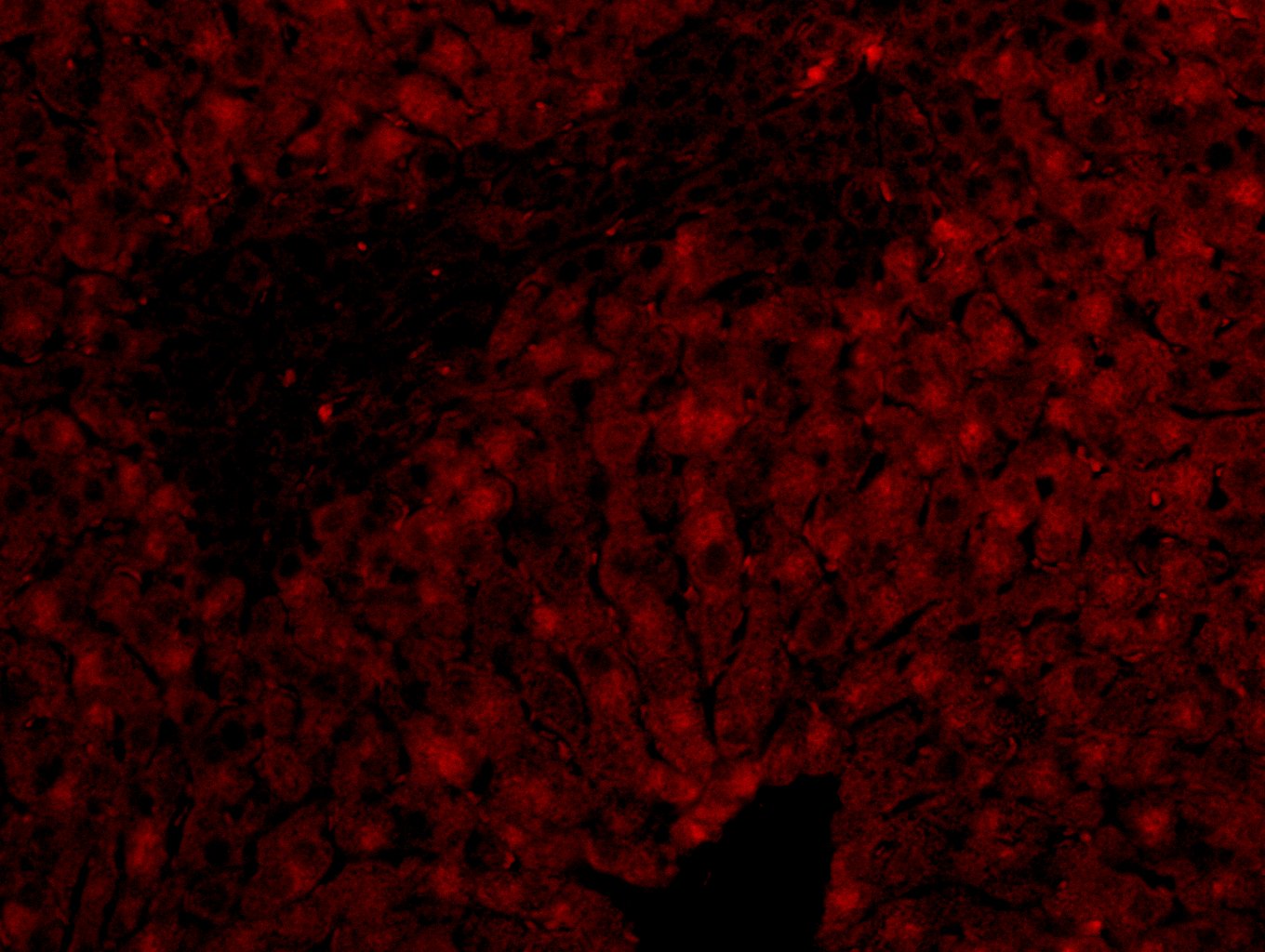

Supplement: Supplemental Information 5 [file peerj-09-12138-s005.zip › Fig 3A/GCS siRNA+ConA GCS.jpg]

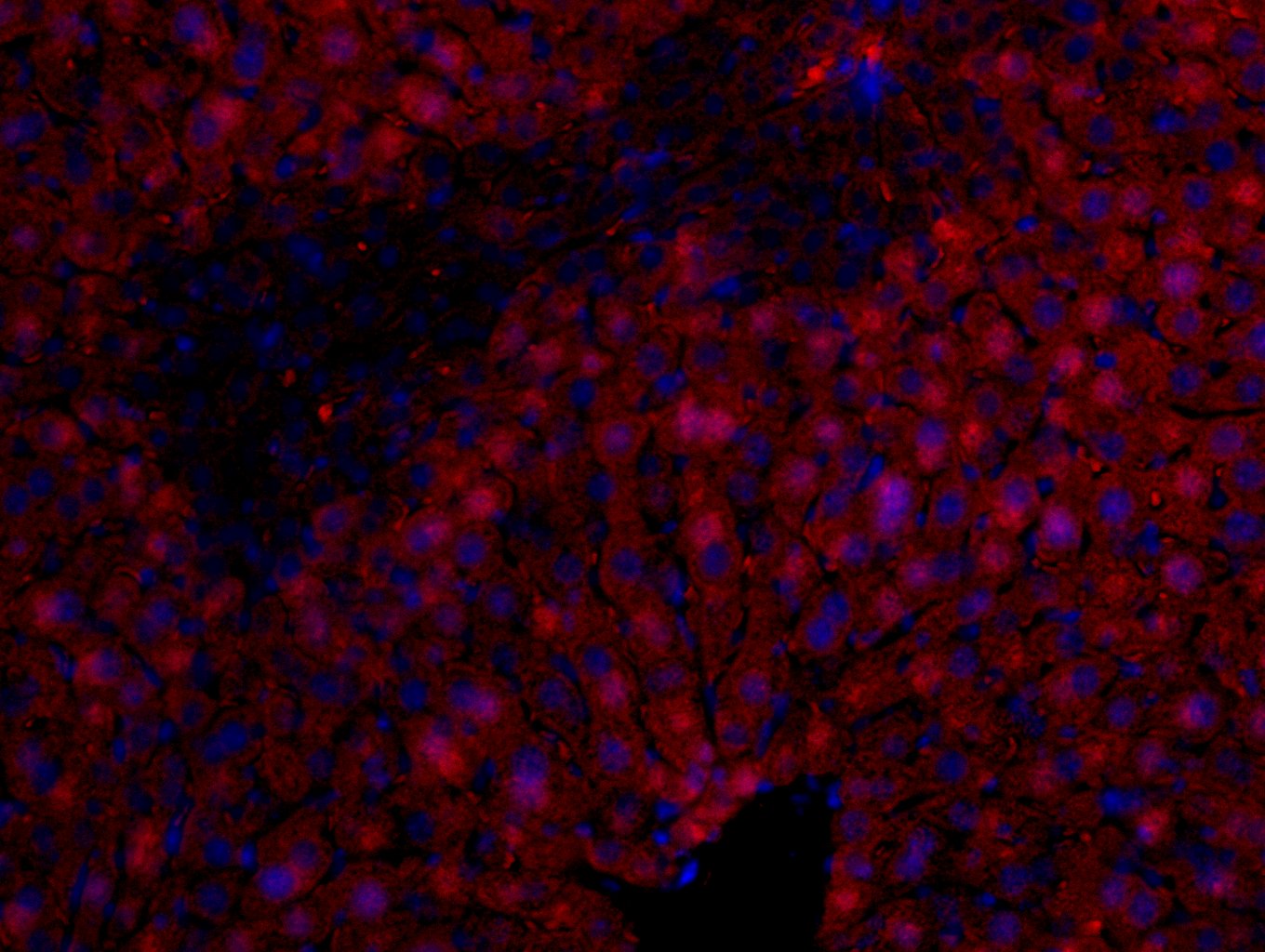

Supplement: Supplemental Information 5 [file peerj-09-12138-s005.zip › Fig 3A/GCS siRNA+ConA merge.jpg]

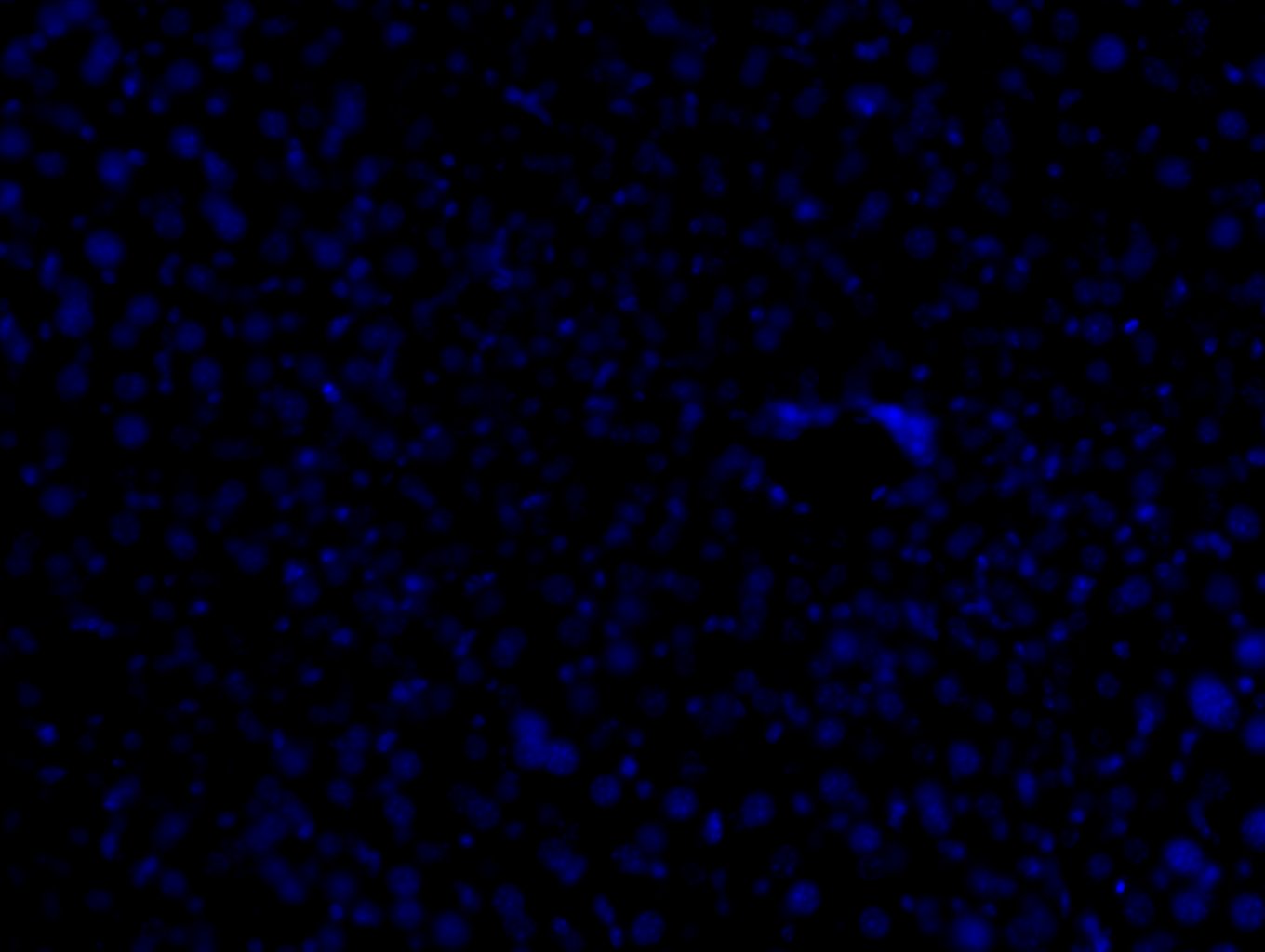

Supplement: Supplemental Information 5 [file peerj-09-12138-s005.zip › Fig 3A/Genz+ConA DAPI.jpg]

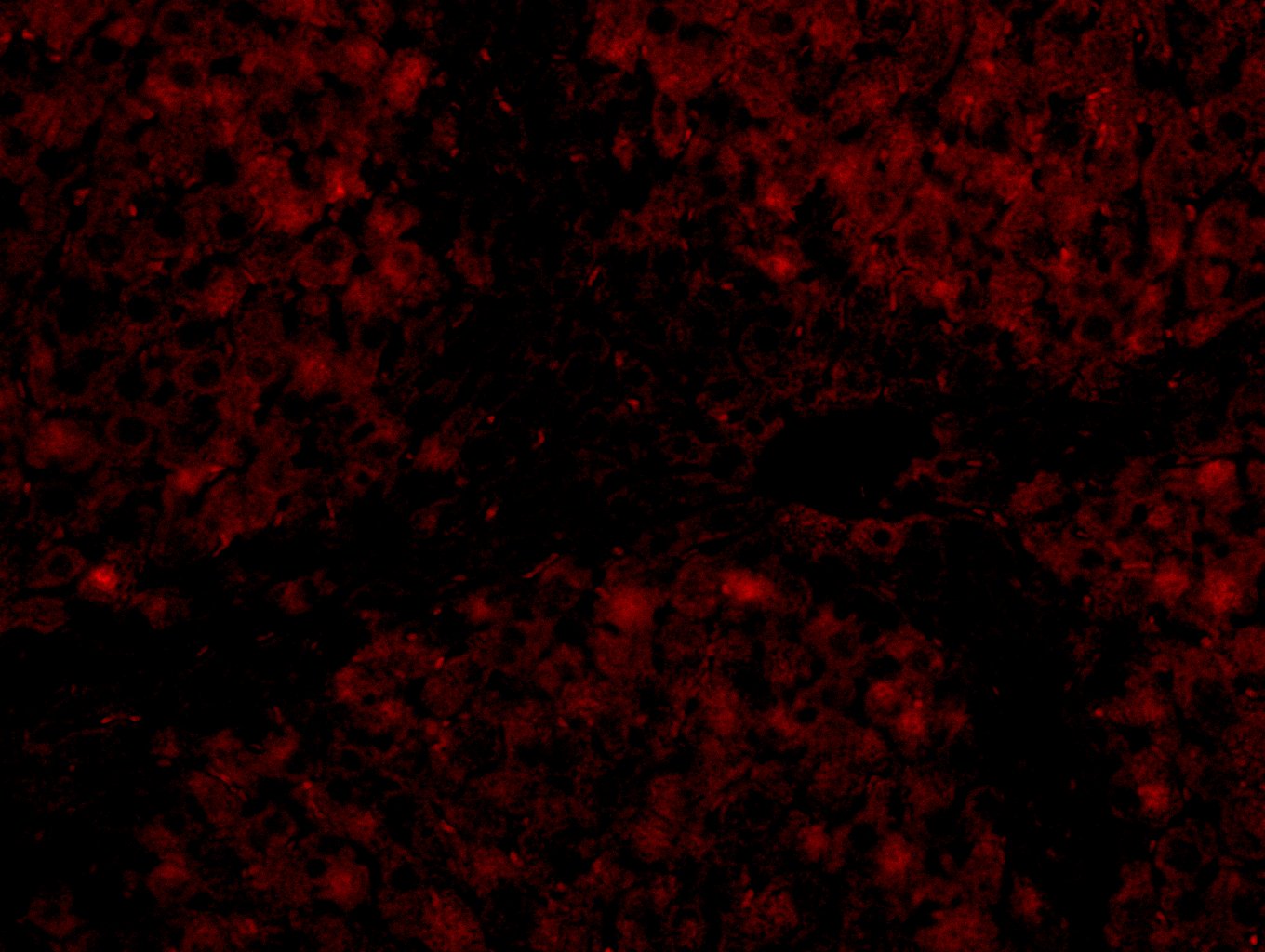

Supplement: Supplemental Information 5 [file peerj-09-12138-s005.zip › Fig 3A/Genz+ConA GCS.jpg]

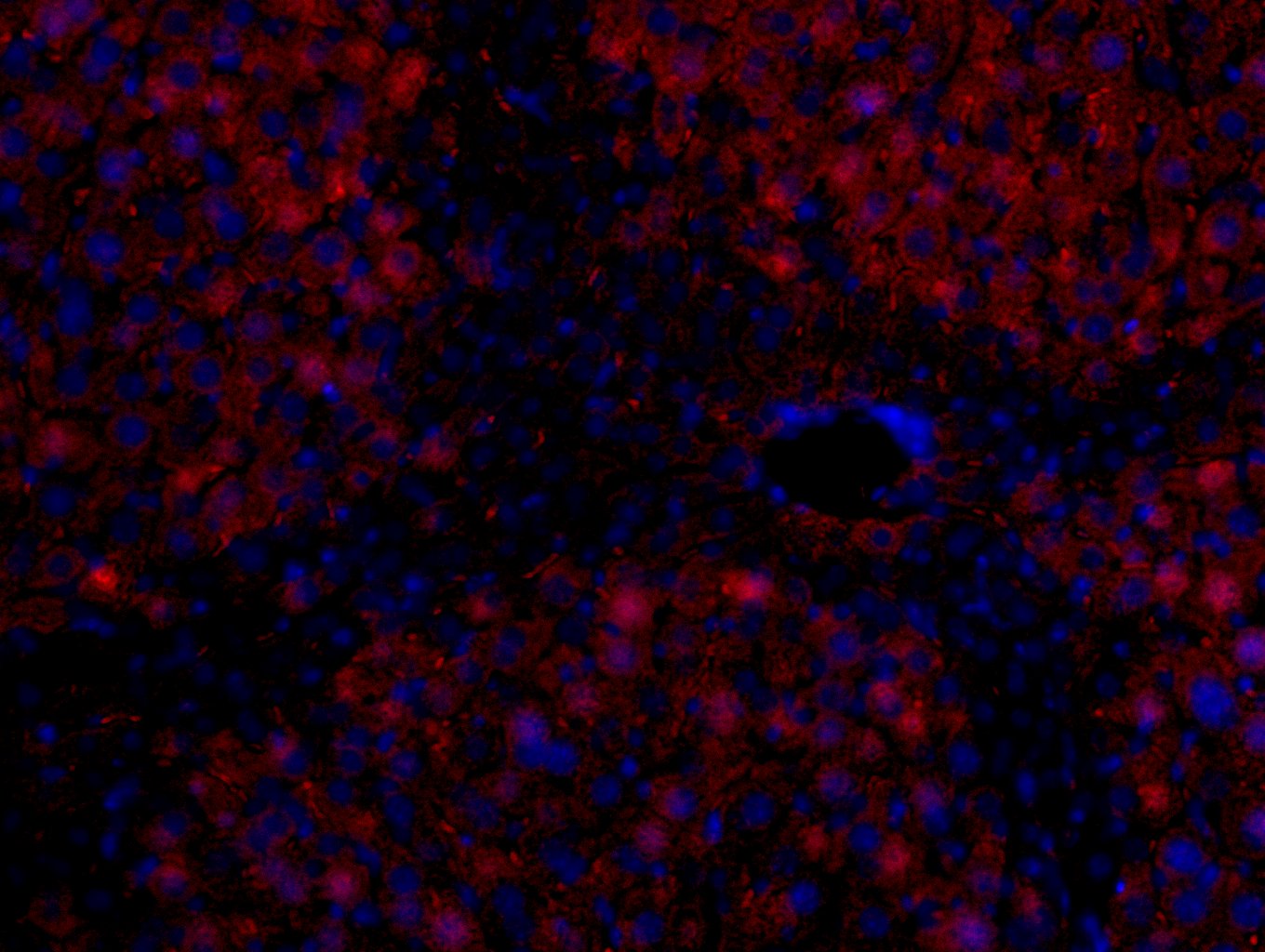

Supplement: Supplemental Information 5 [file peerj-09-12138-s005.zip › Fig 3A/Genz+ConA merge.jpg]

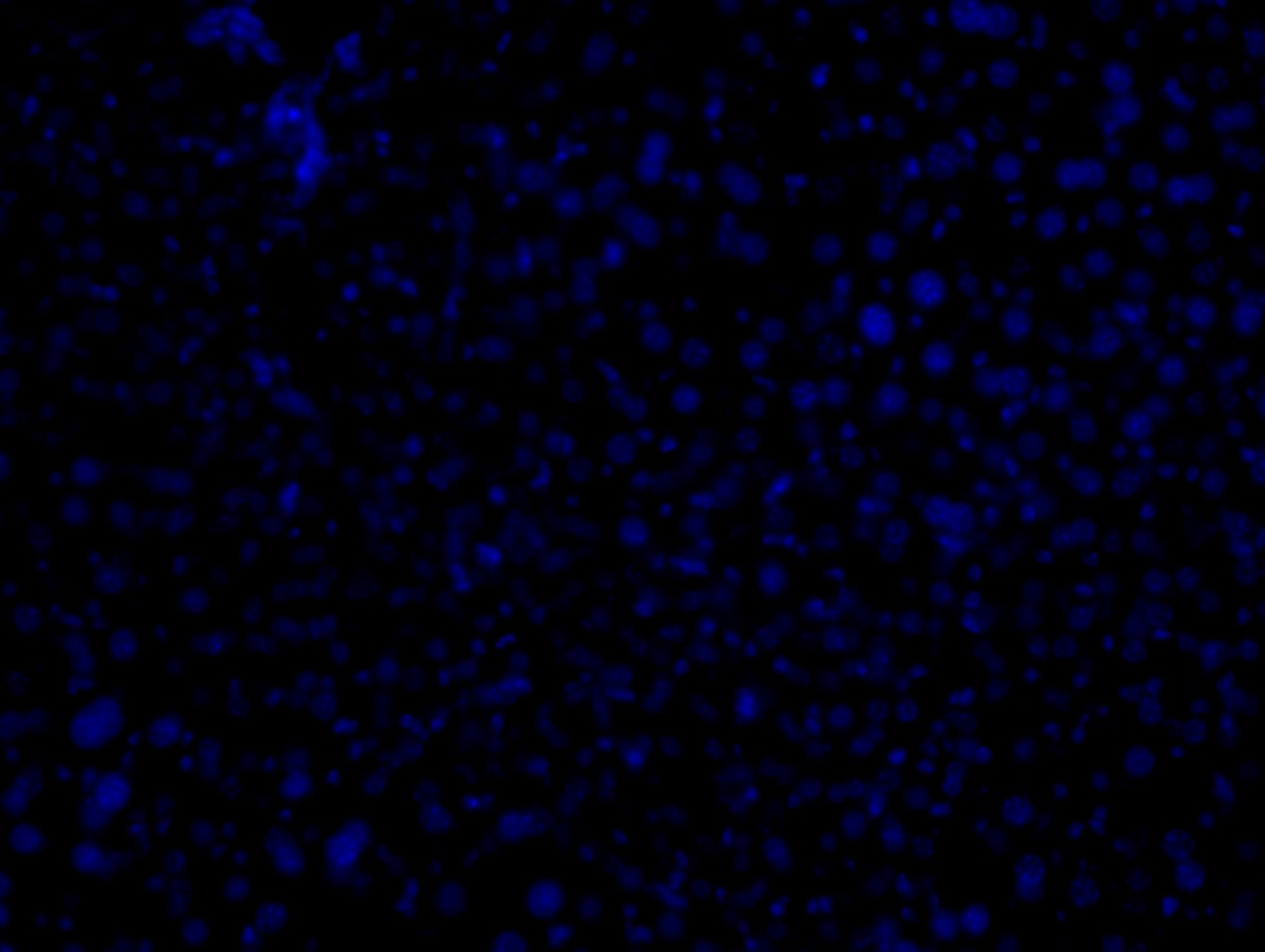

Supplement: Supplemental Information 5 [file peerj-09-12138-s005.zip › Fig 3A/NC DAPI.jpg]

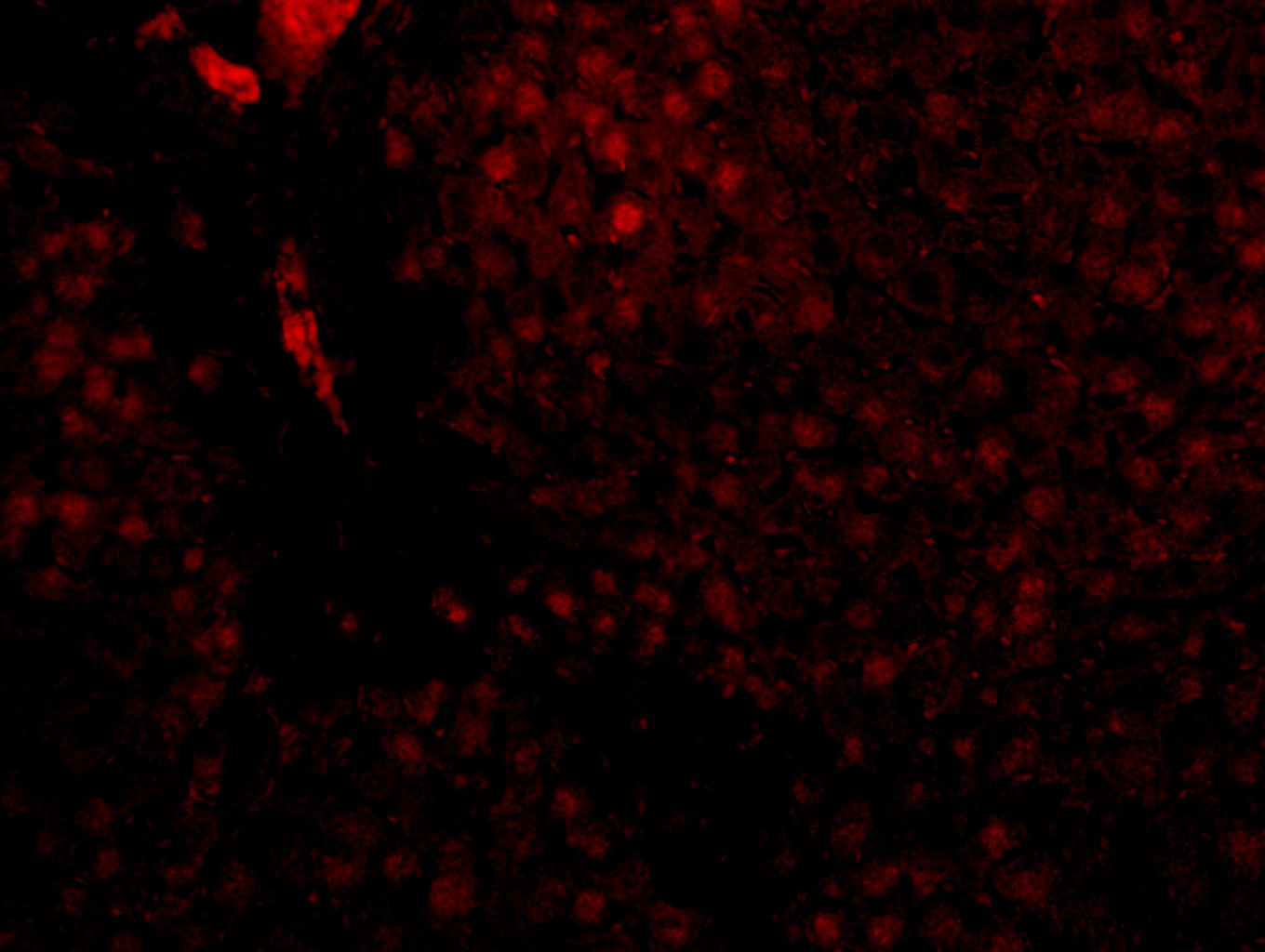

Supplement: Supplemental Information 5 [file peerj-09-12138-s005.zip › Fig 3A/NC GCS.jpg]

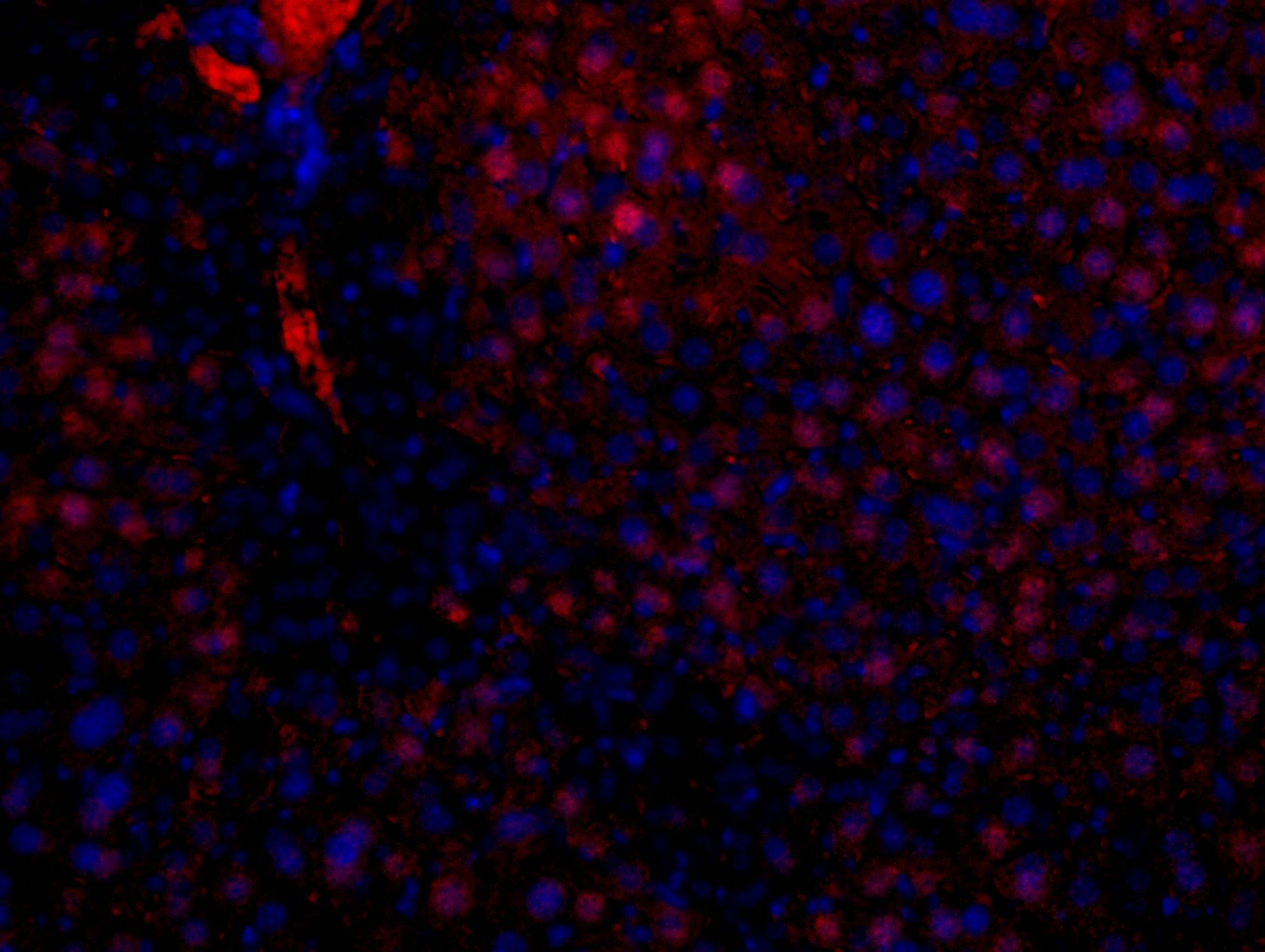

Supplement: Supplemental Information 5 [file peerj-09-12138-s005.zip › Fig 3A/NC merge.jpg]

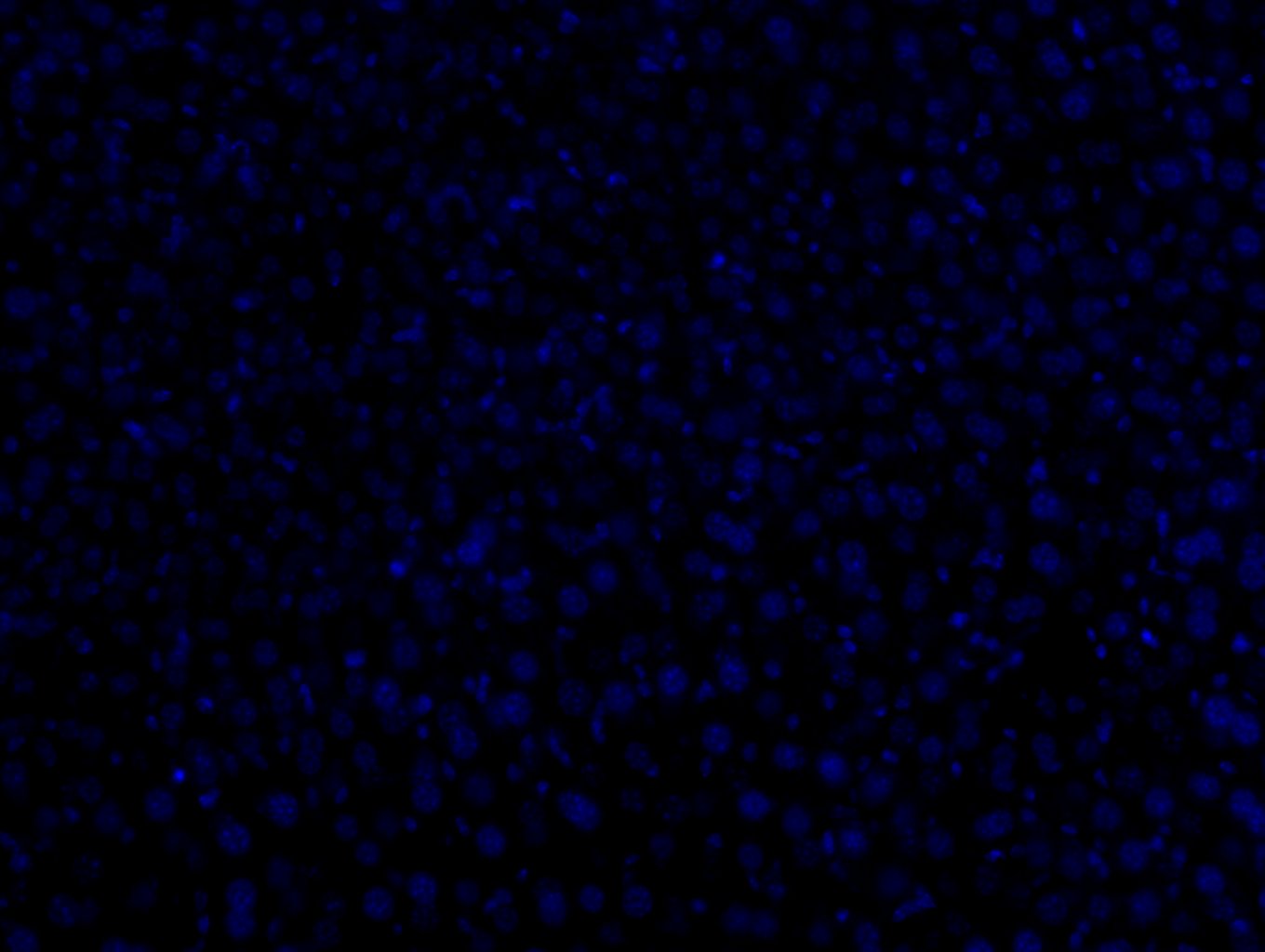

Supplement: Supplemental Information 5 [file peerj-09-12138-s005.zip › Fig 3A/NT DAPI.jpg]

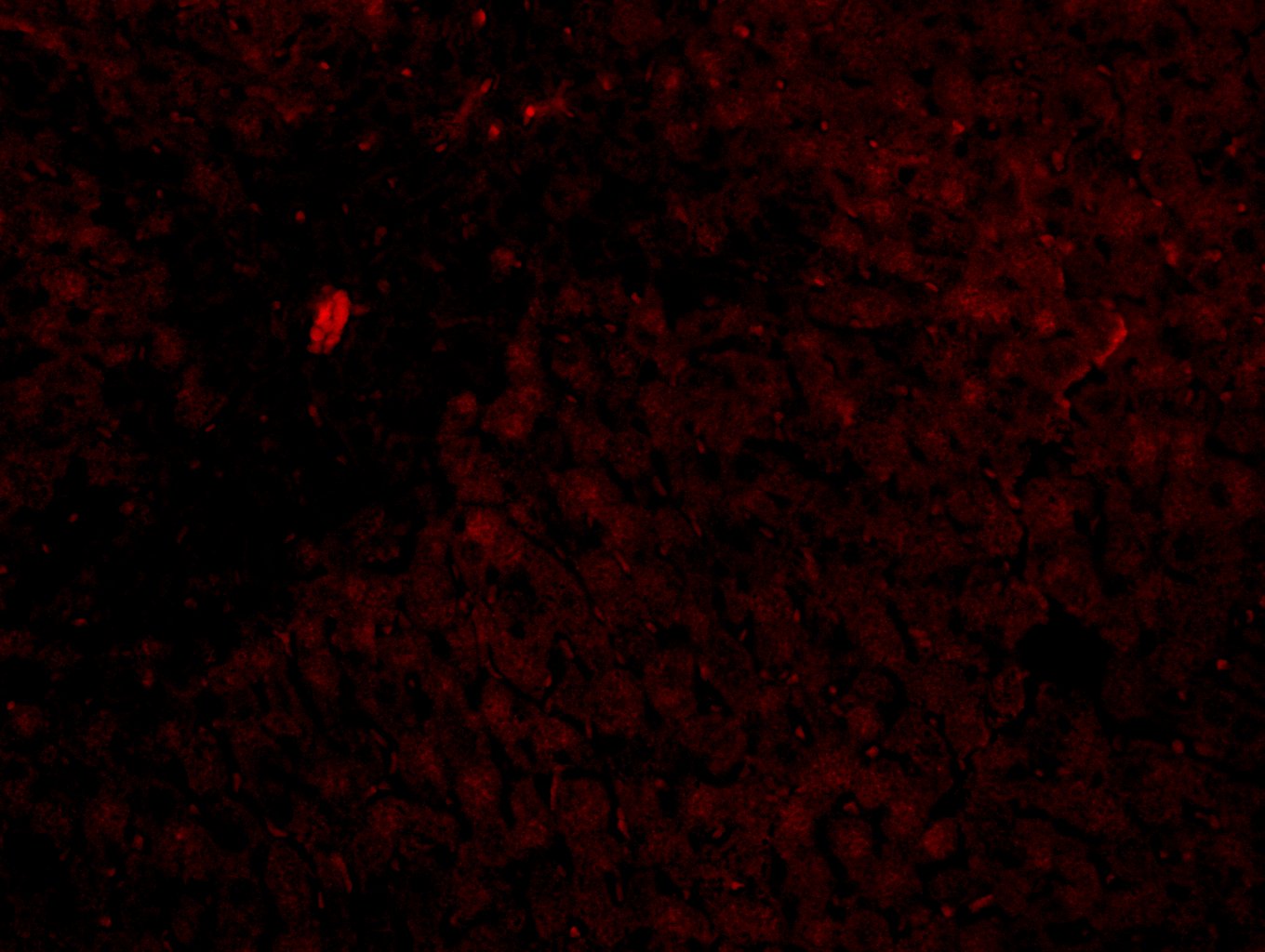

Supplement: Supplemental Information 5 [file peerj-09-12138-s005.zip › Fig 3A/NT GCS.jpg]

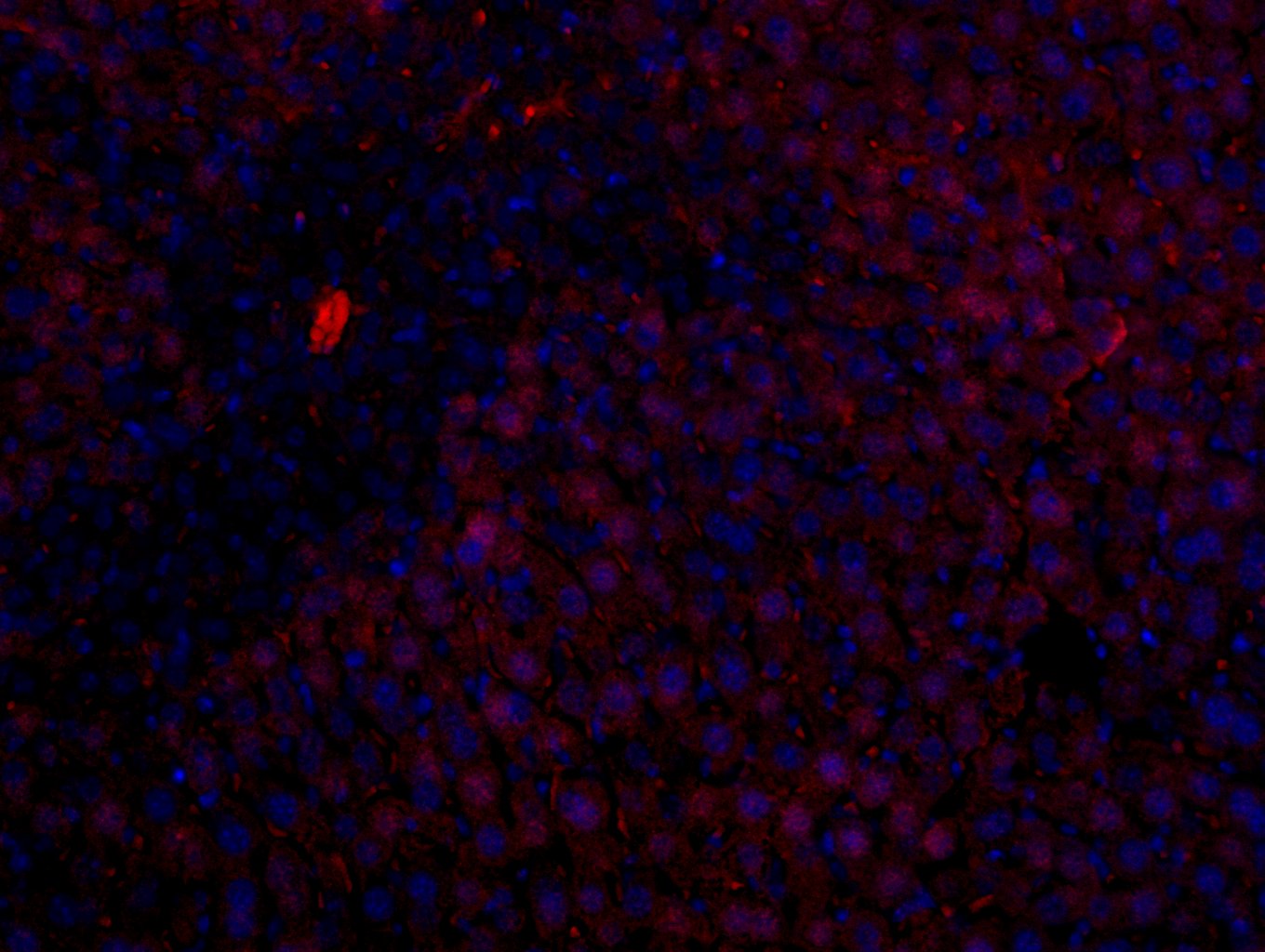

Supplement: Supplemental Information 5 [file peerj-09-12138-s005.zip › Fig 3A/NT merge.jpg]

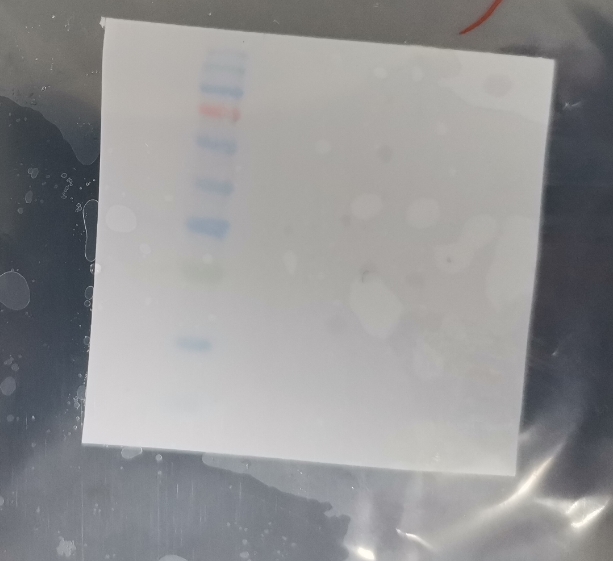

Supplement: Supplemental Information 5 [file peerj-09-12138-s005.zip › Fig 3B/GAPDH.jpg]

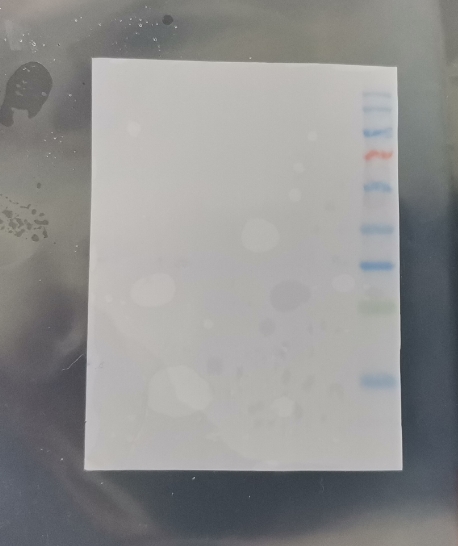

Supplement: Supplemental Information 5 [file peerj-09-12138-s005.zip › Fig 3B/GCS-01.jpg]

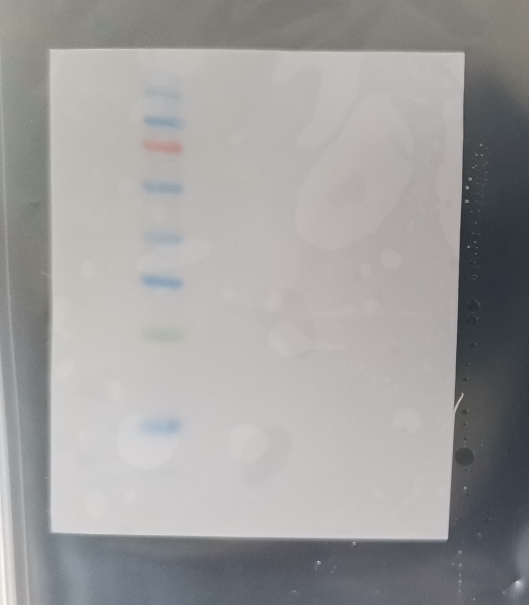

Supplement: Supplemental Information 5 [file peerj-09-12138-s005.zip › Fig 3B/GCS-02.jpg]

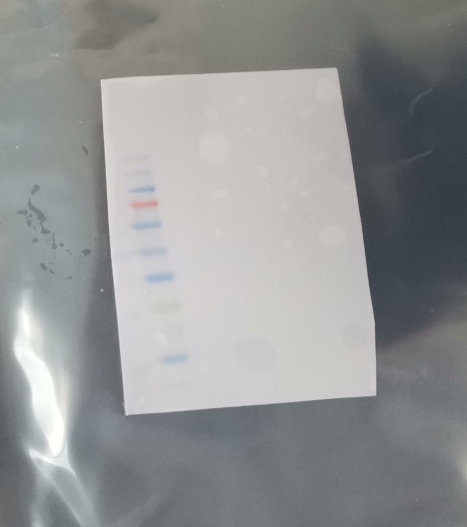

Supplement: Supplemental Information 5 [file peerj-09-12138-s005.zip › Fig 3B/GCS-03.jpg]

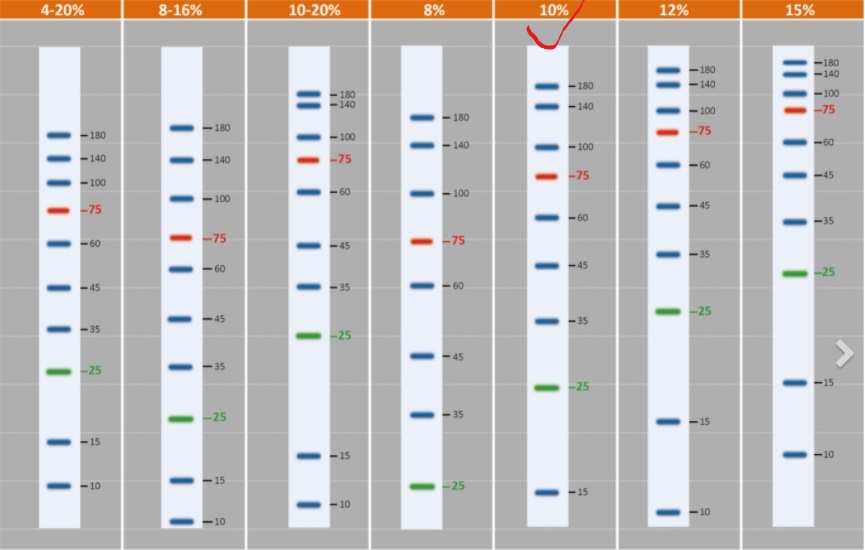

Supplement: Supplemental Information 5 [file peerj-09-12138-s005.zip › Fig 3B/protein marker.jpg]

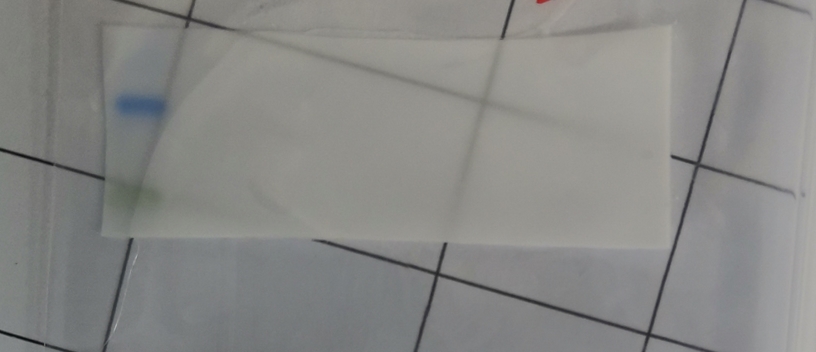

Supplement: Supplemental Information 5 [file peerj-09-12138-s005.zip › Fig 4C/cleaved-caspase3-01.jpg]

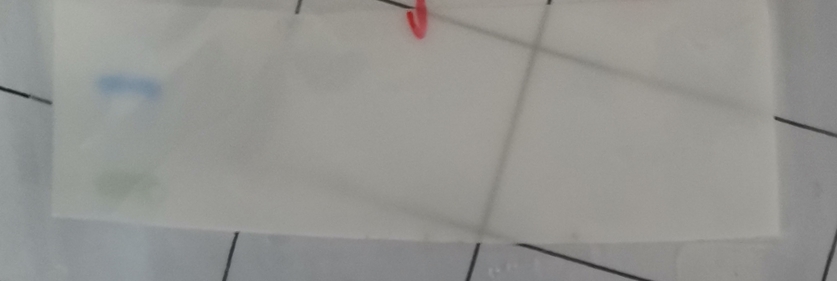

Supplement: Supplemental Information 5 [file peerj-09-12138-s005.zip › Fig 4C/cleaved-caspase3-02.jpg]

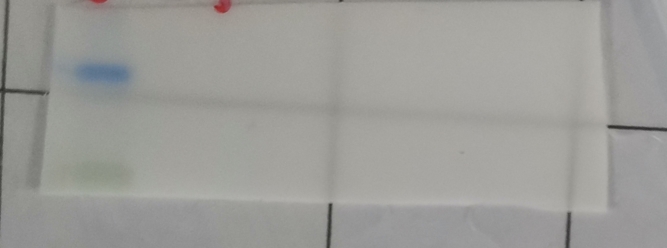

Supplement: Supplemental Information 5 [file peerj-09-12138-s005.zip › Fig 4C/cleaved-caspase3-03.jpg]

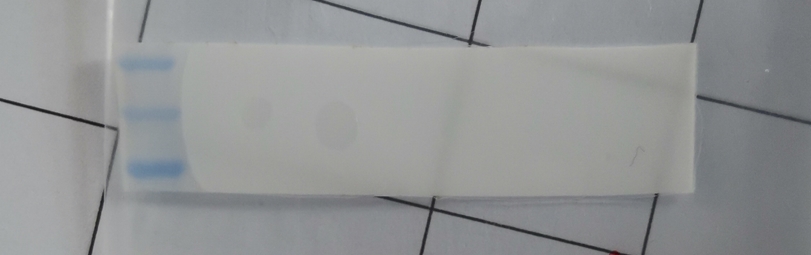

Supplement: Supplemental Information 5 [file peerj-09-12138-s005.zip › Fig 4C/GAPDH.jpg]

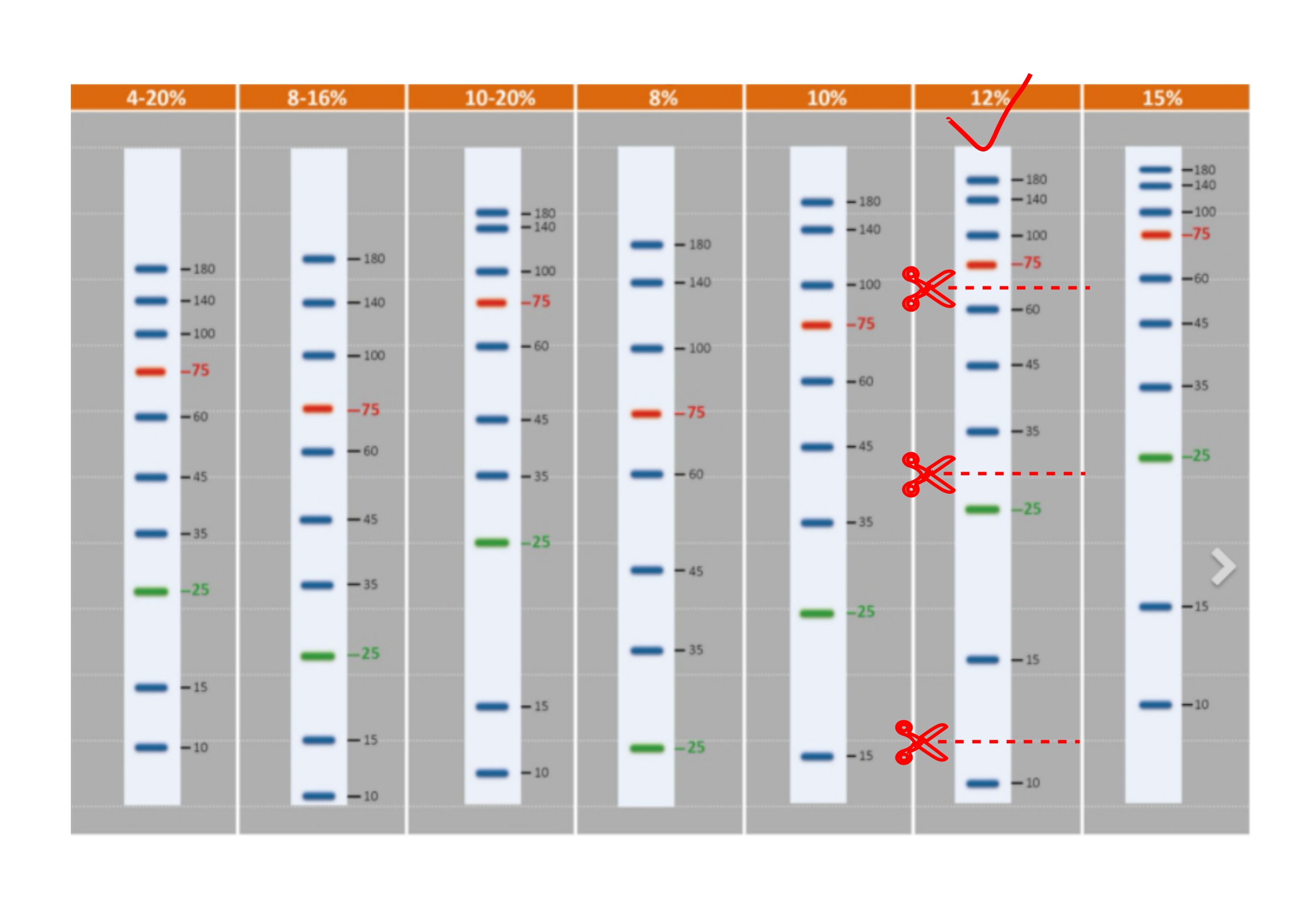

Supplement: Supplemental Information 5 [file peerj-09-12138-s005.zip › Fig 4C/protein marker.jpg]

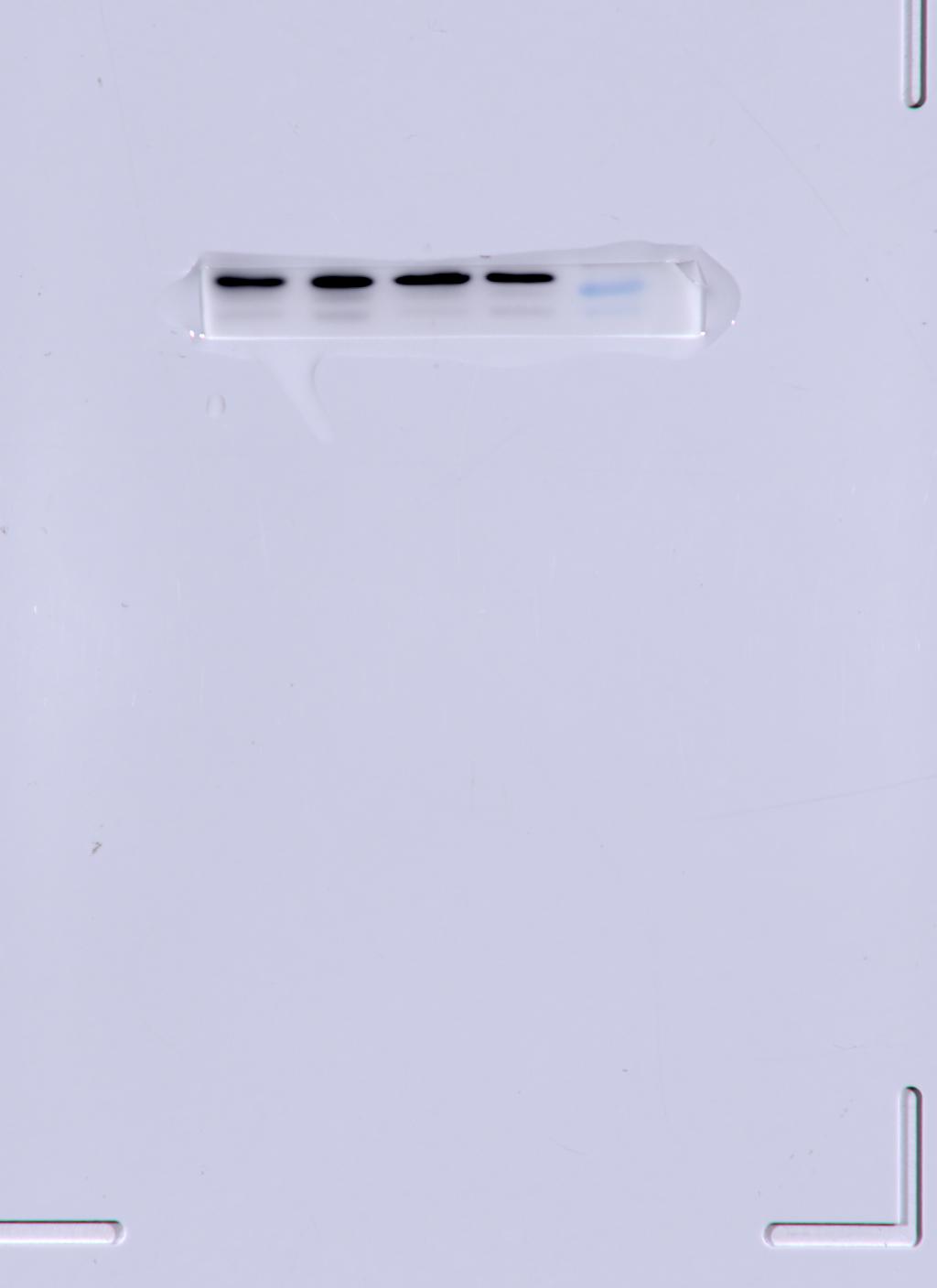

Supplement: Supplemental Information 5 [file peerj-09-12138-s005.zip › Fig 6C/WB-1/GAPDH-01.jpg]

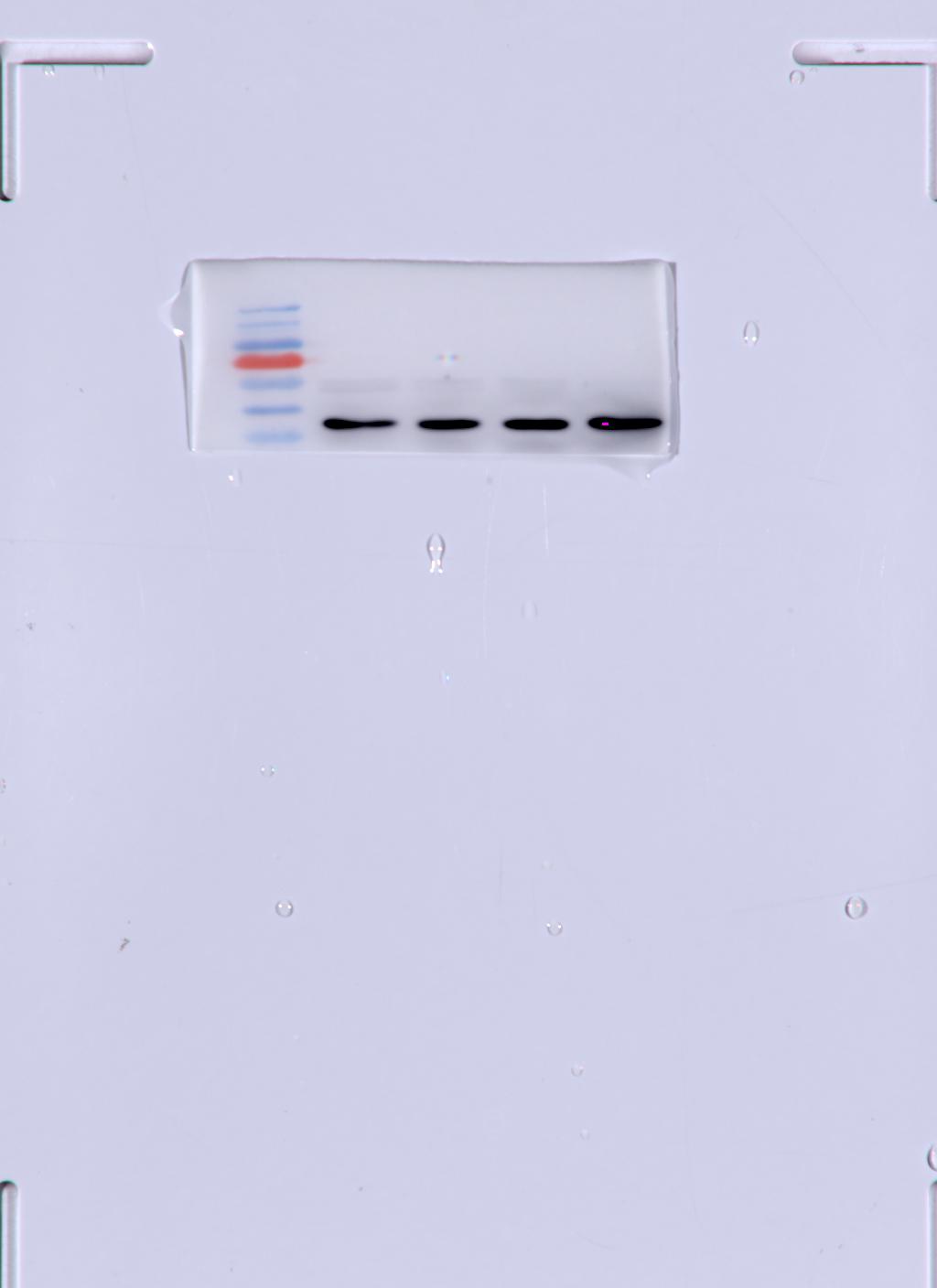

Supplement: Supplemental Information 5 [file peerj-09-12138-s005.zip › Fig 6C/WB-1/GAPDH-02.jpg]

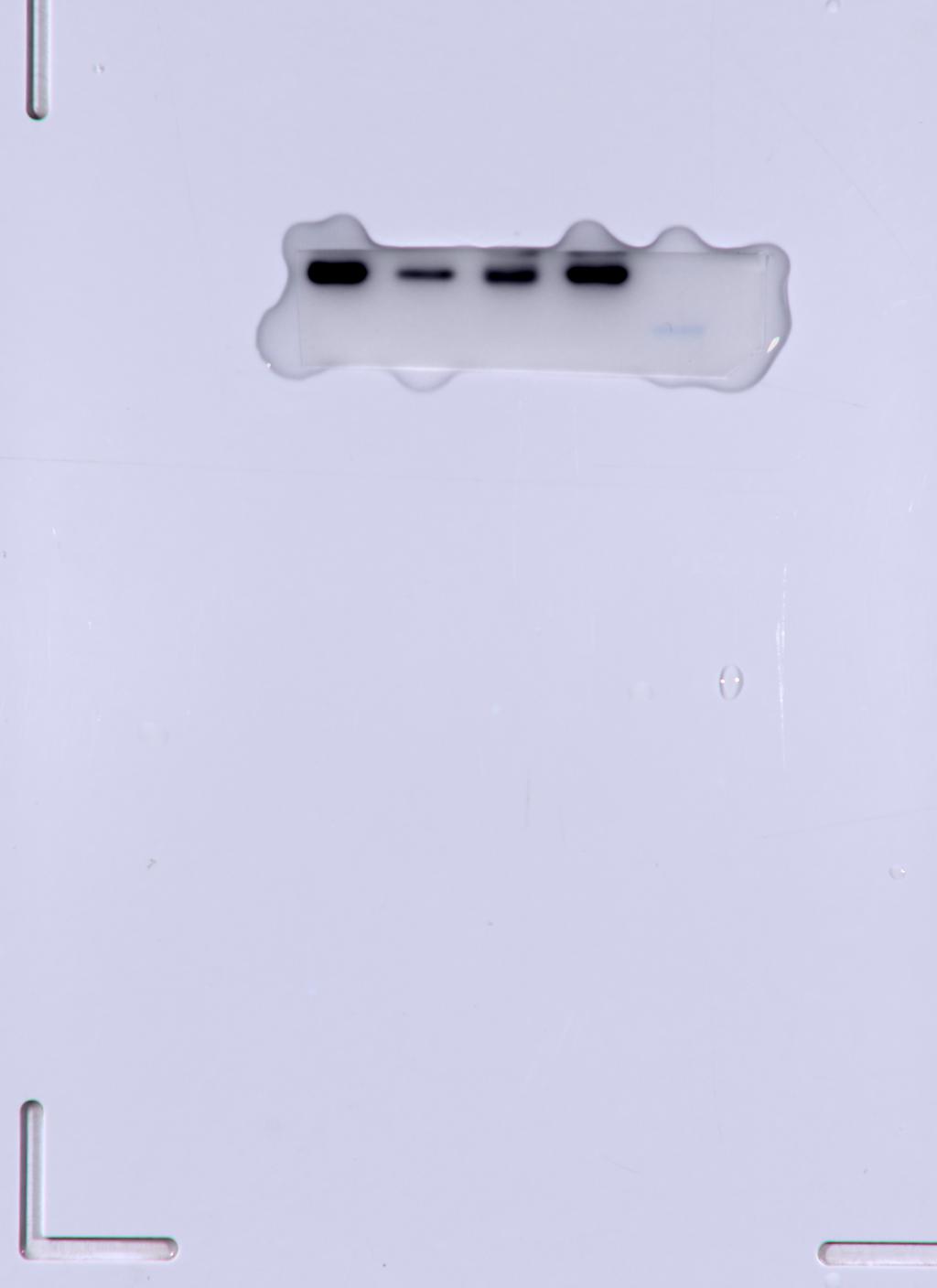

Supplement: Supplemental Information 5 [file peerj-09-12138-s005.zip › Fig 6C/WB-1/mmp-01.jpg]

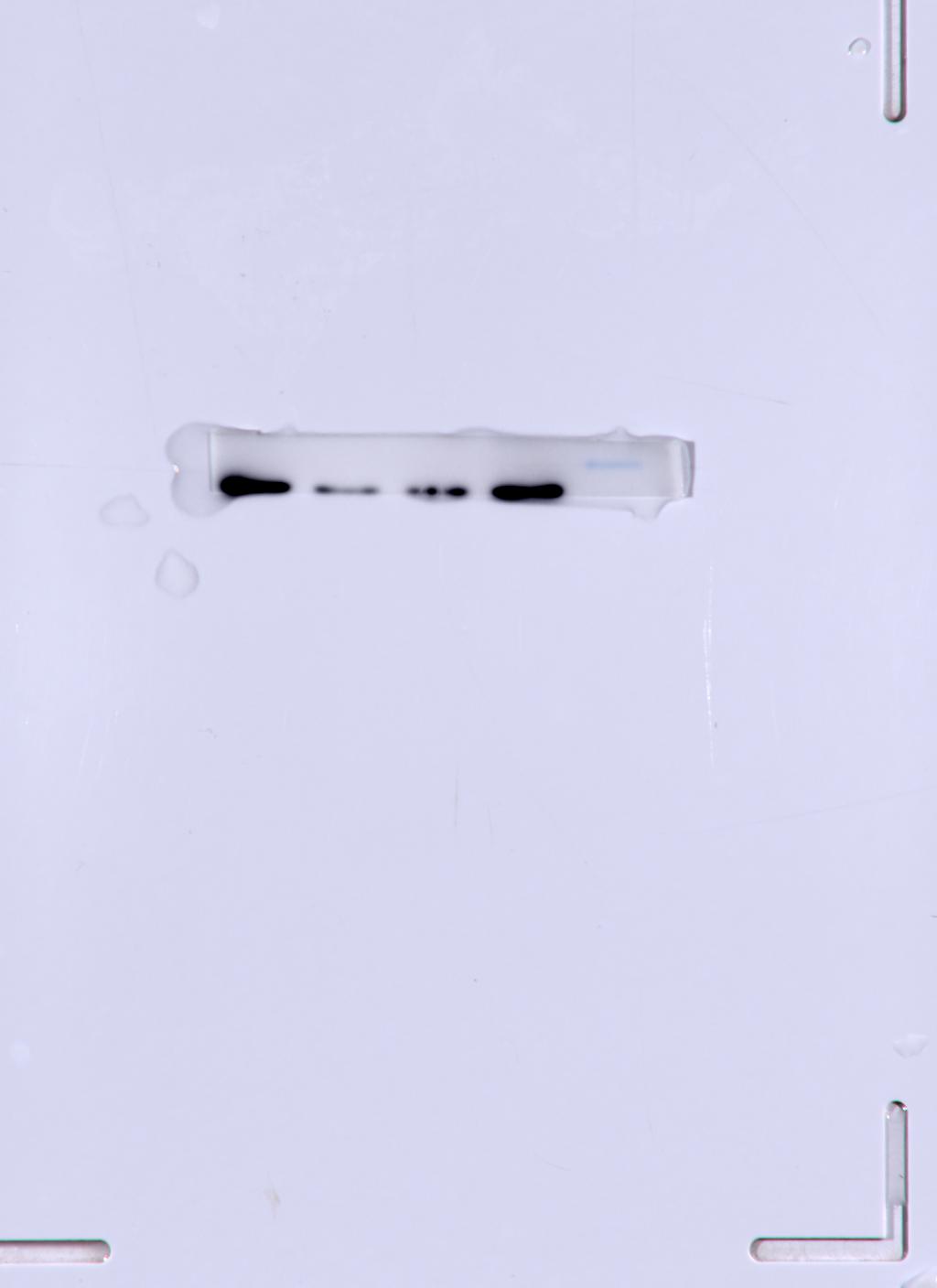

Supplement: Supplemental Information 5 [file peerj-09-12138-s005.zip › Fig 6C/WB-1/mmp-02.jpg]

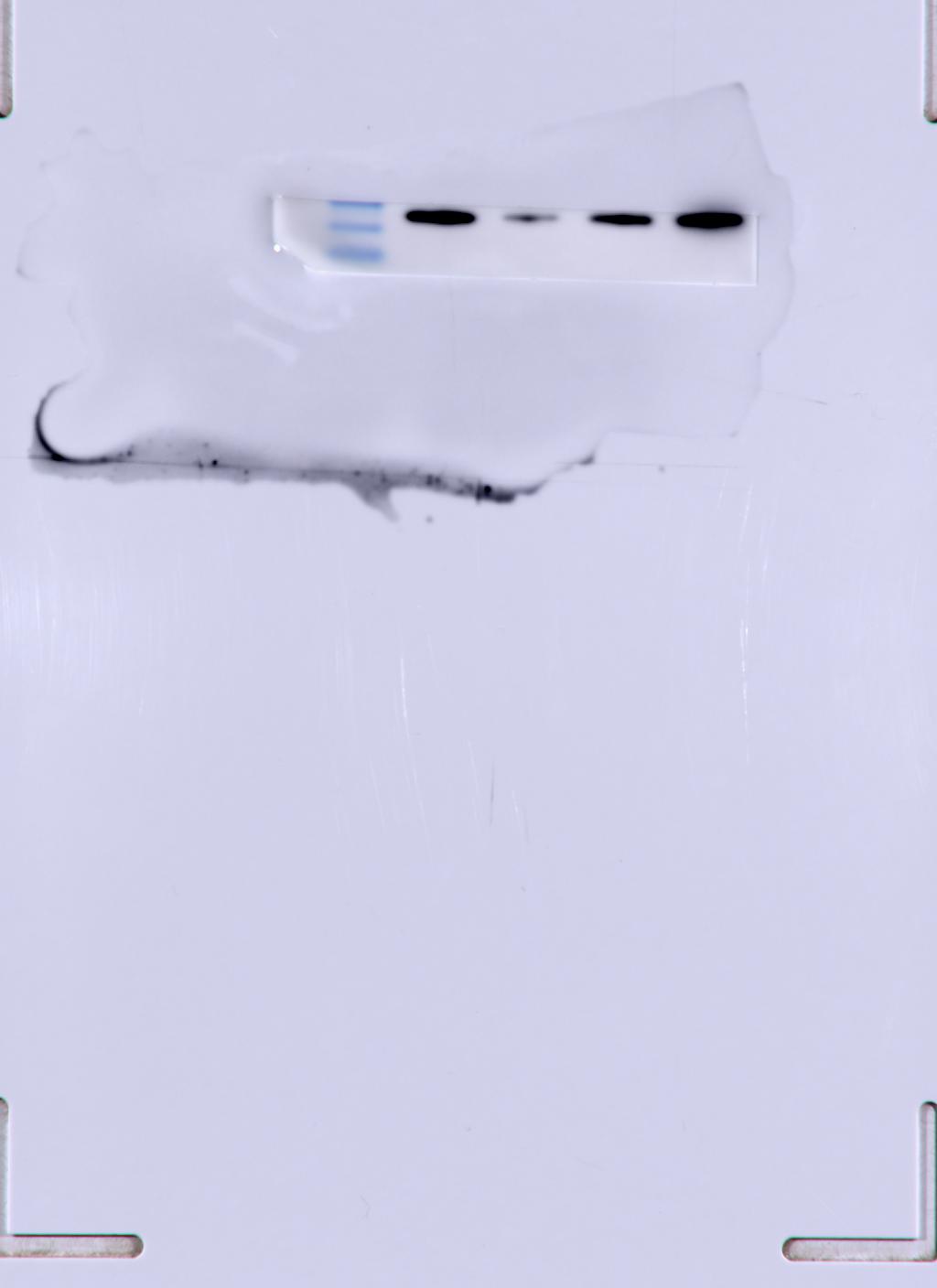

Supplement: Supplemental Information 5 [file peerj-09-12138-s005.zip › Fig 6C/WB-1/mmp-03.jpg]

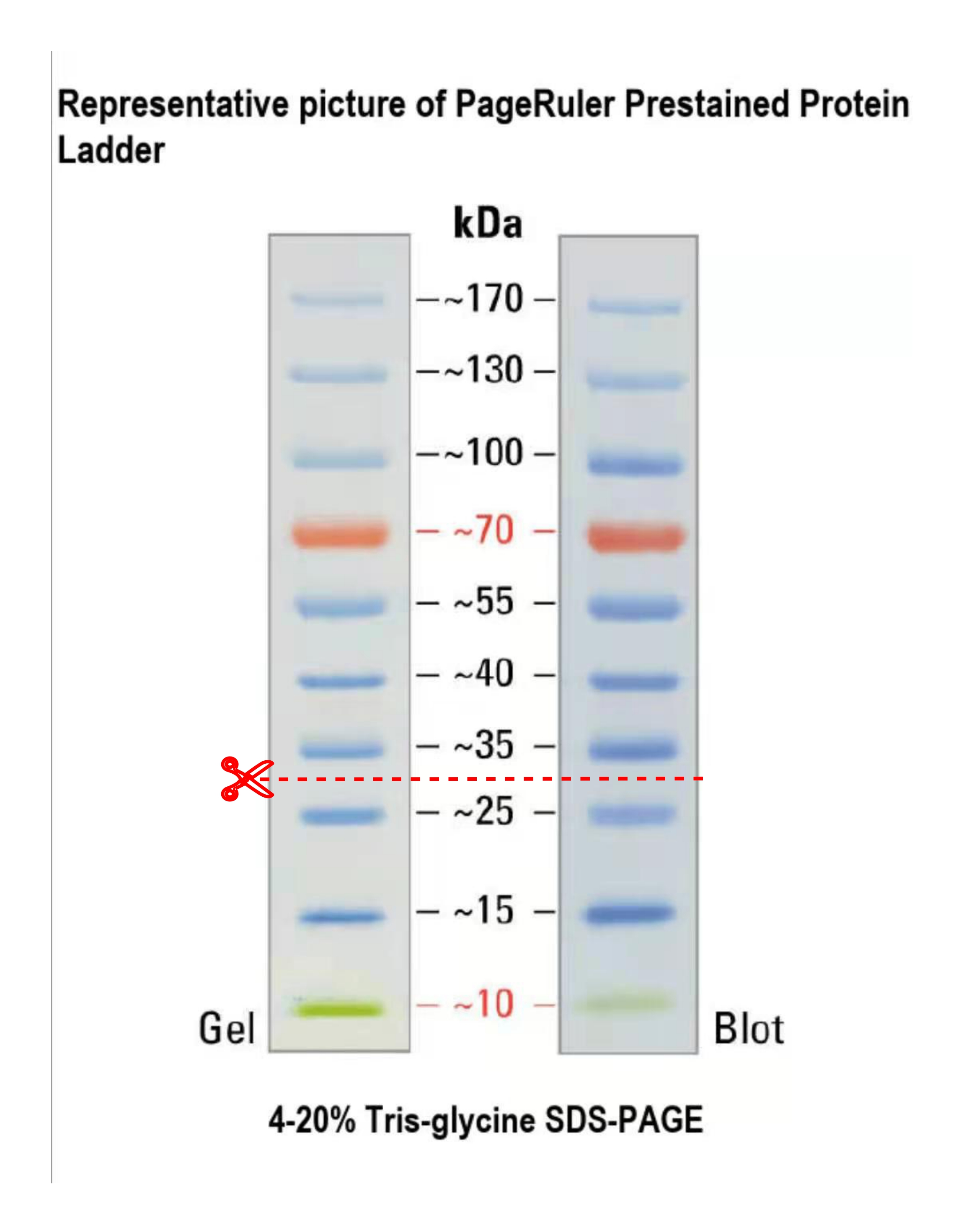

Supplement: Supplemental Information 5 [file peerj-09-12138-s005.zip › Fig 6C/WB-1/protein marker.jpg]

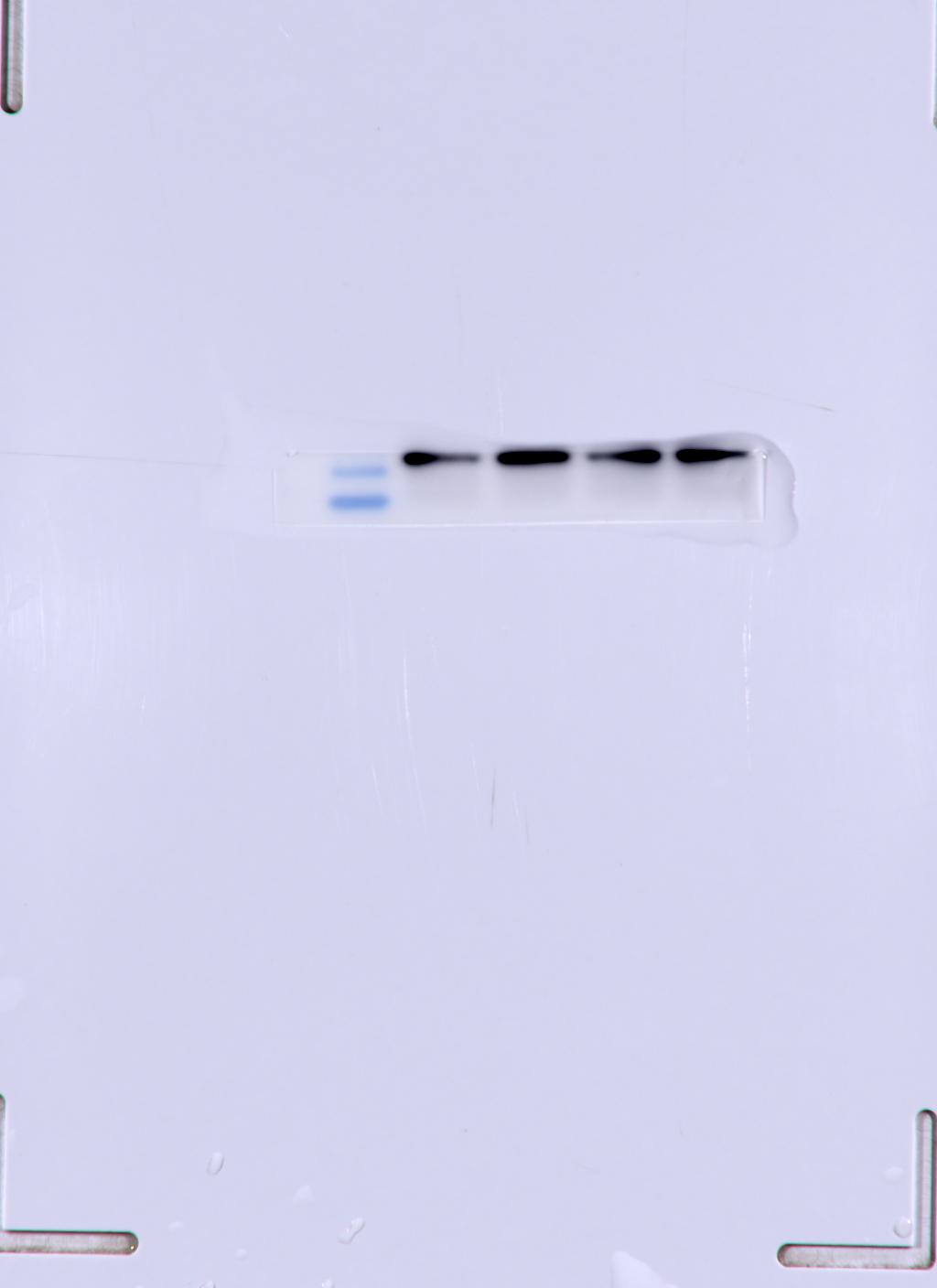

Supplement: Supplemental Information 5 [file peerj-09-12138-s005.zip › Fig 6C/WB-1/timp-01.jpg]

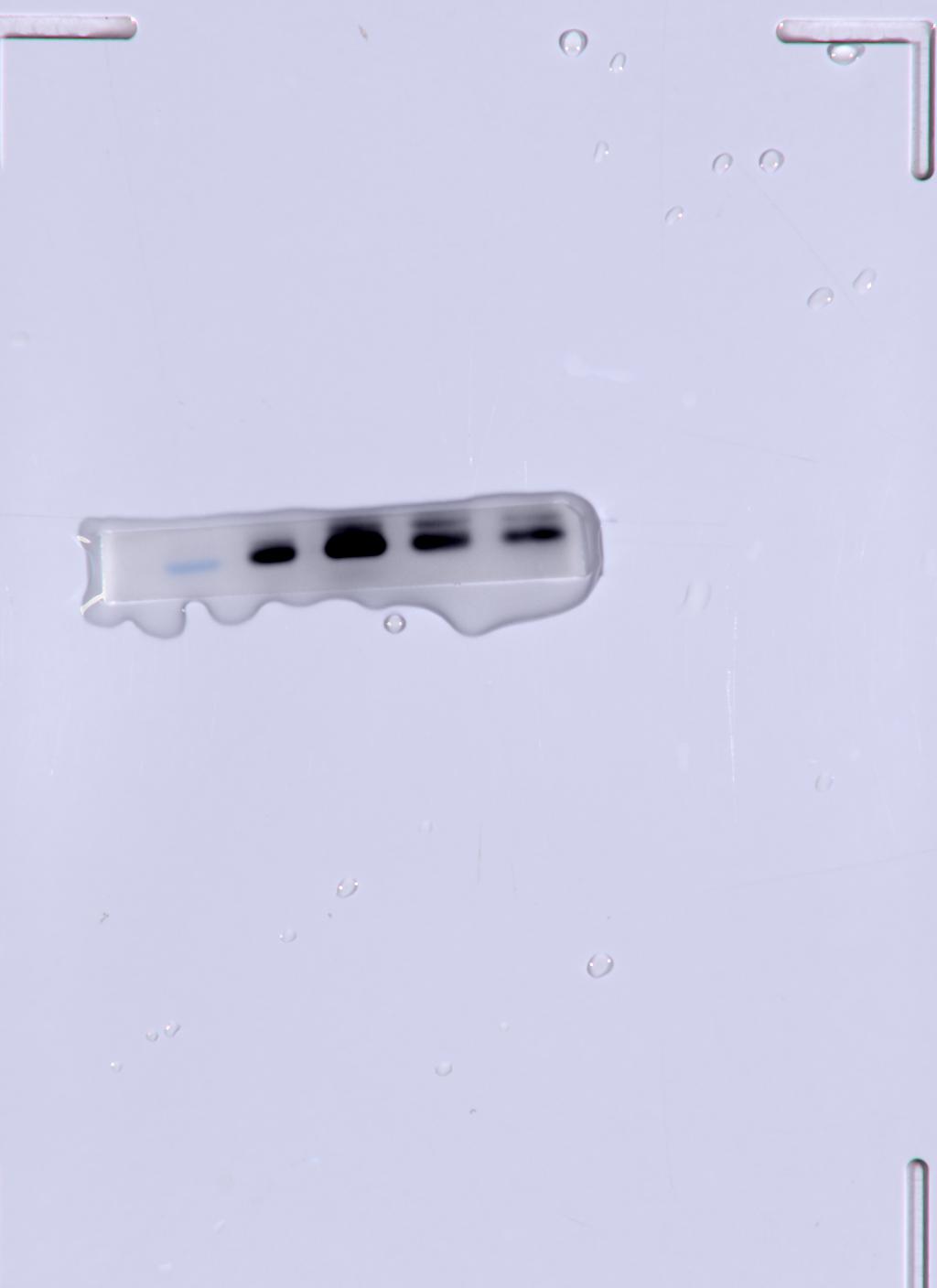

Supplement: Supplemental Information 5 [file peerj-09-12138-s005.zip › Fig 6C/WB-1/timp-02.jpg]

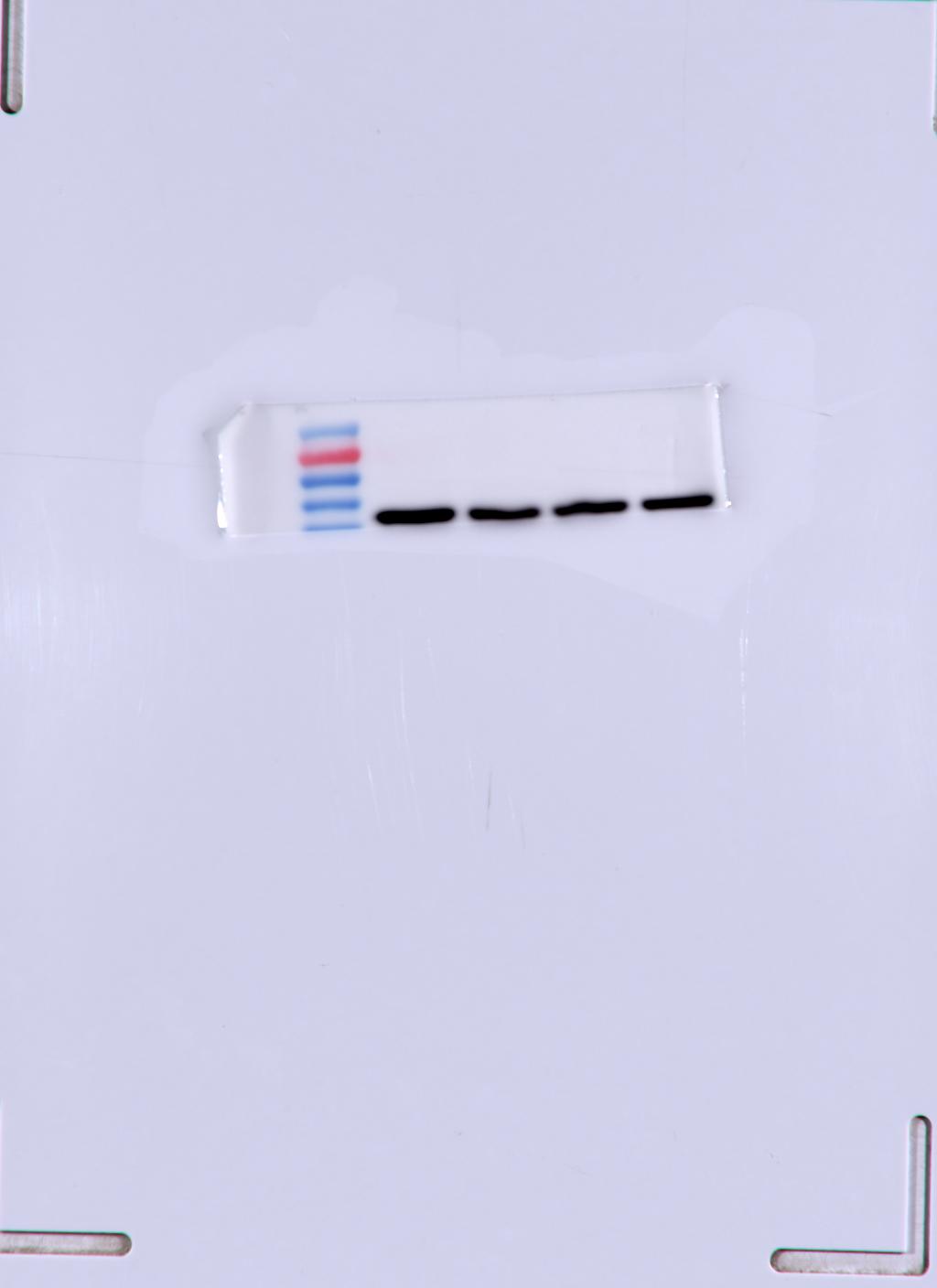

Supplement: Supplemental Information 5 [file peerj-09-12138-s005.zip › Fig 6C/WB-2/GAPDH-01.jpg]

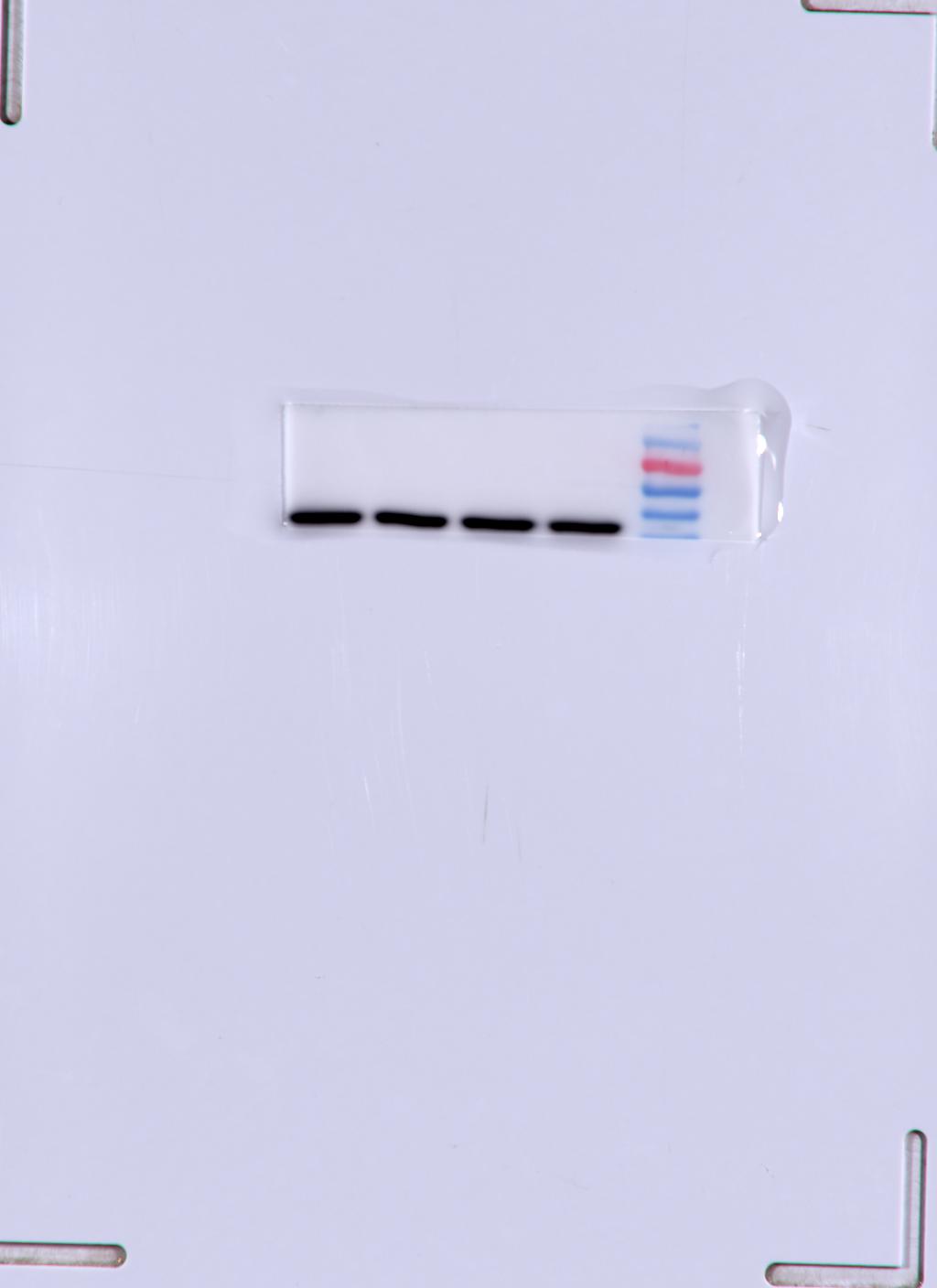

Supplement: Supplemental Information 5 [file peerj-09-12138-s005.zip › Fig 6C/WB-2/GAPDH-02.jpg]

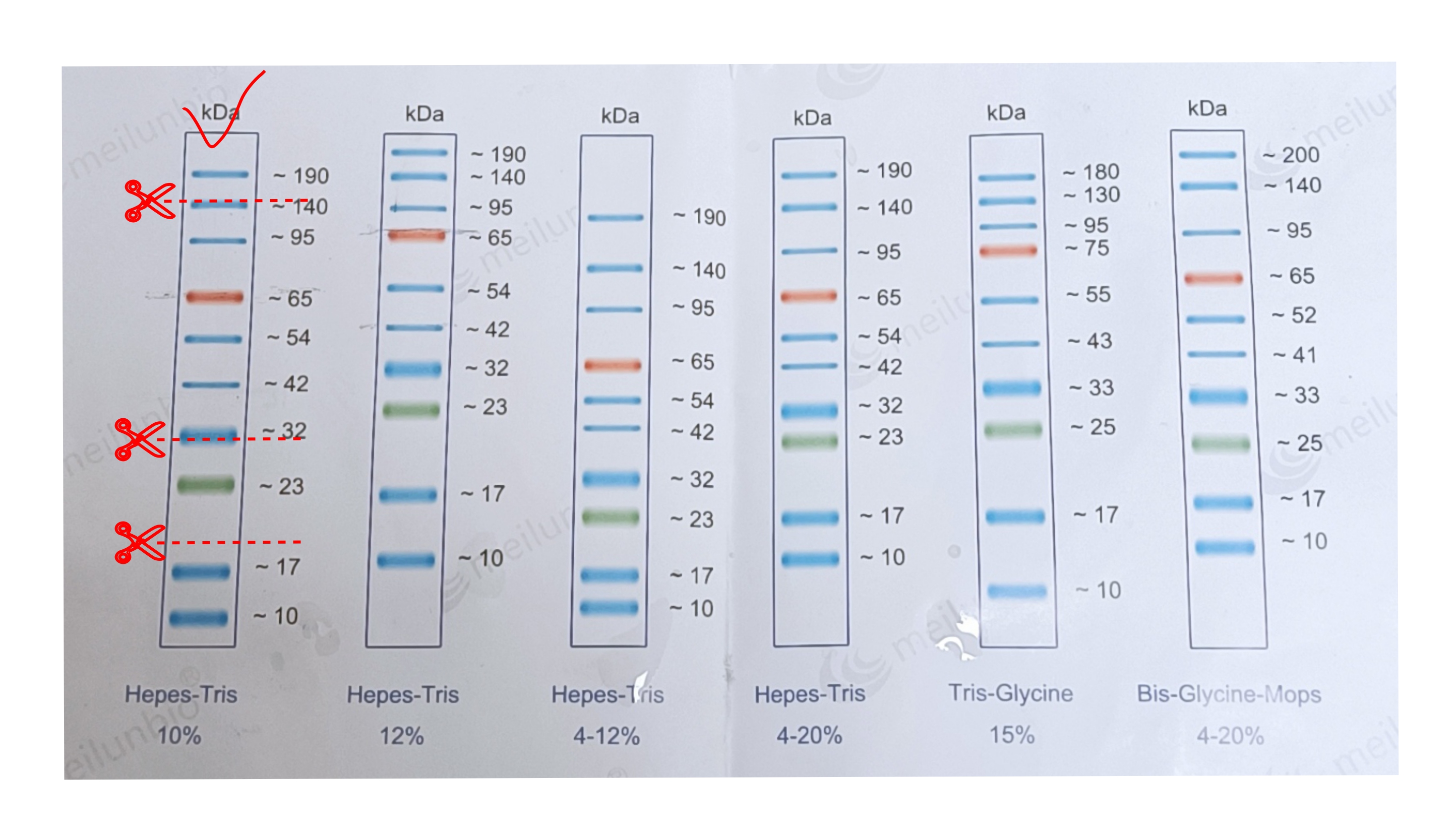

Supplement: Supplemental Information 5 [file peerj-09-12138-s005.zip › Fig 6C/WB-2/protein marker.jpg]

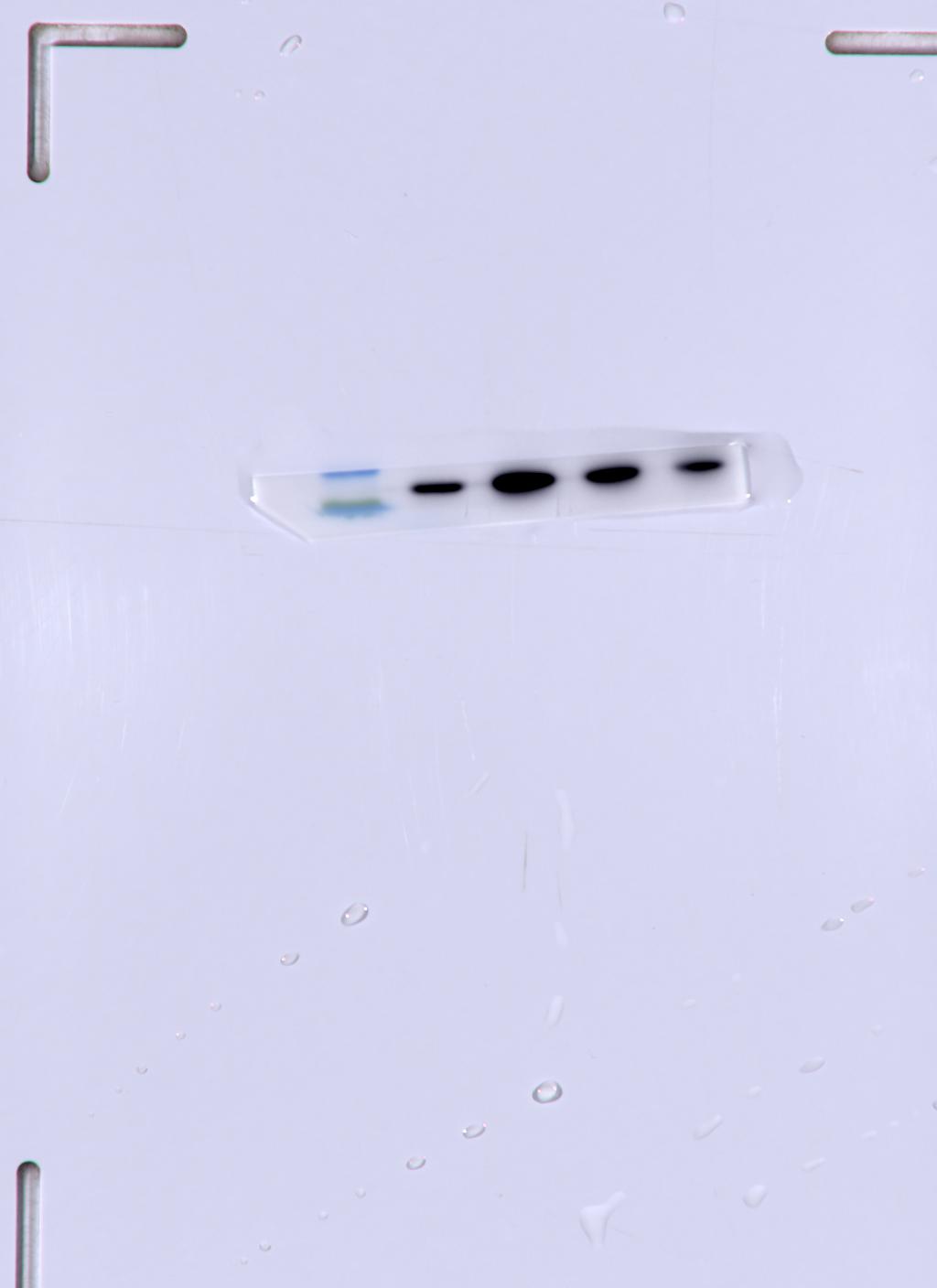

Supplement: Supplemental Information 5 [file peerj-09-12138-s005.zip › Fig 6C/WB-2/timp-01.jpg]

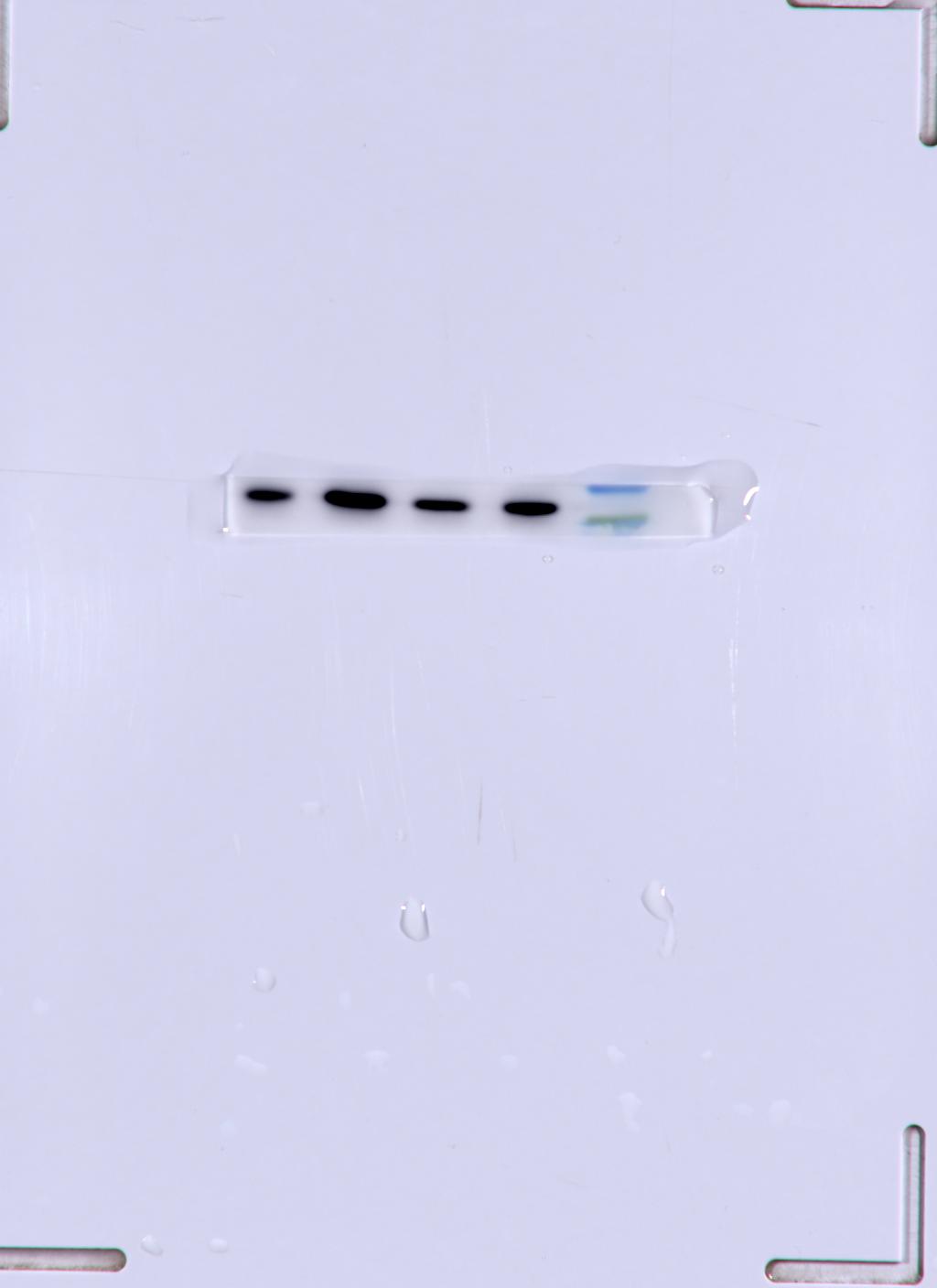

Supplement: Supplemental Information 5 [file peerj-09-12138-s005.zip › Fig 6C/WB-2/timp-02.jpg]

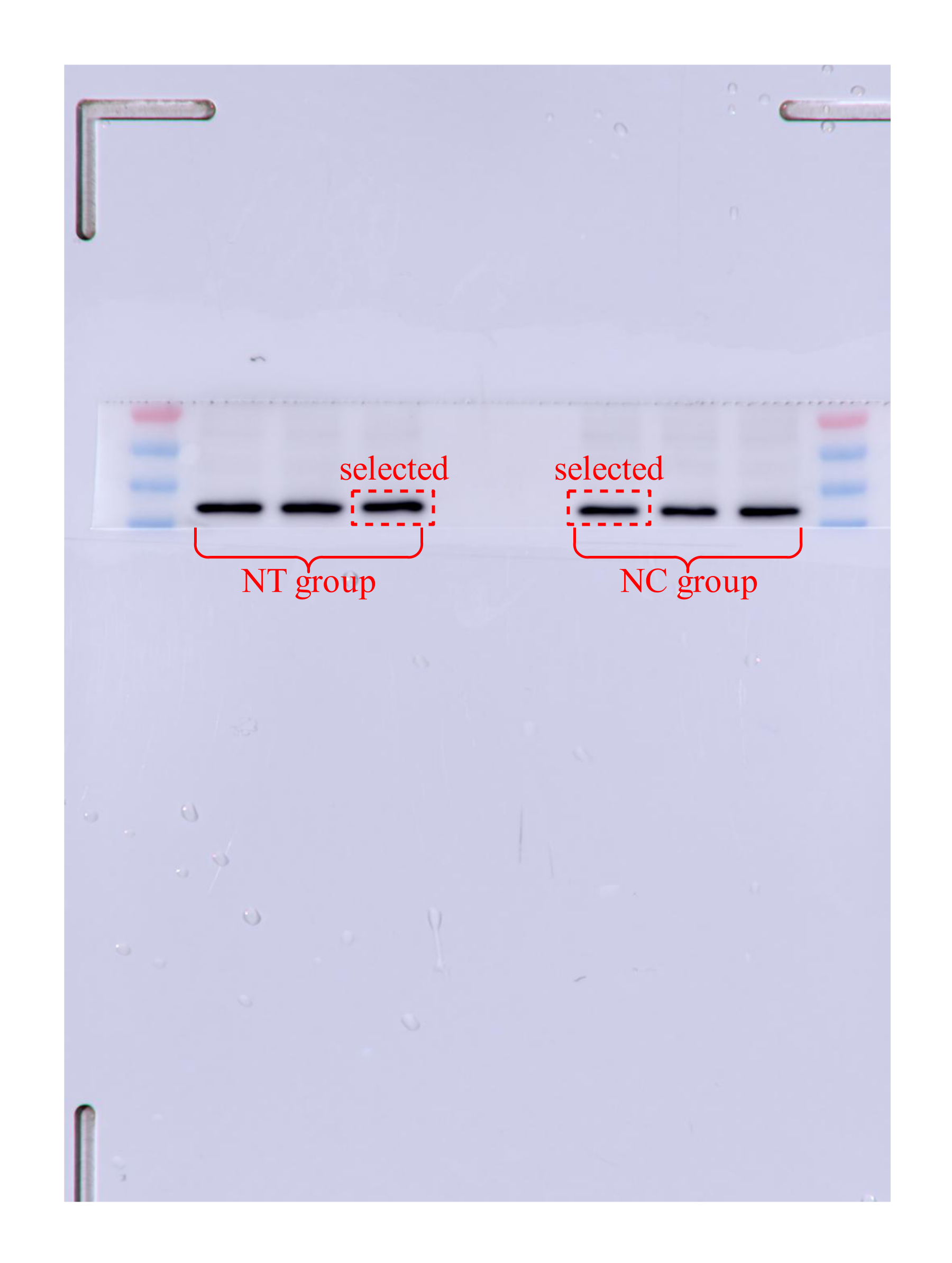

Supplement: Supplemental Information 5 [file peerj-09-12138-s005.zip › Fig 6D/GAPDH grouped.png]

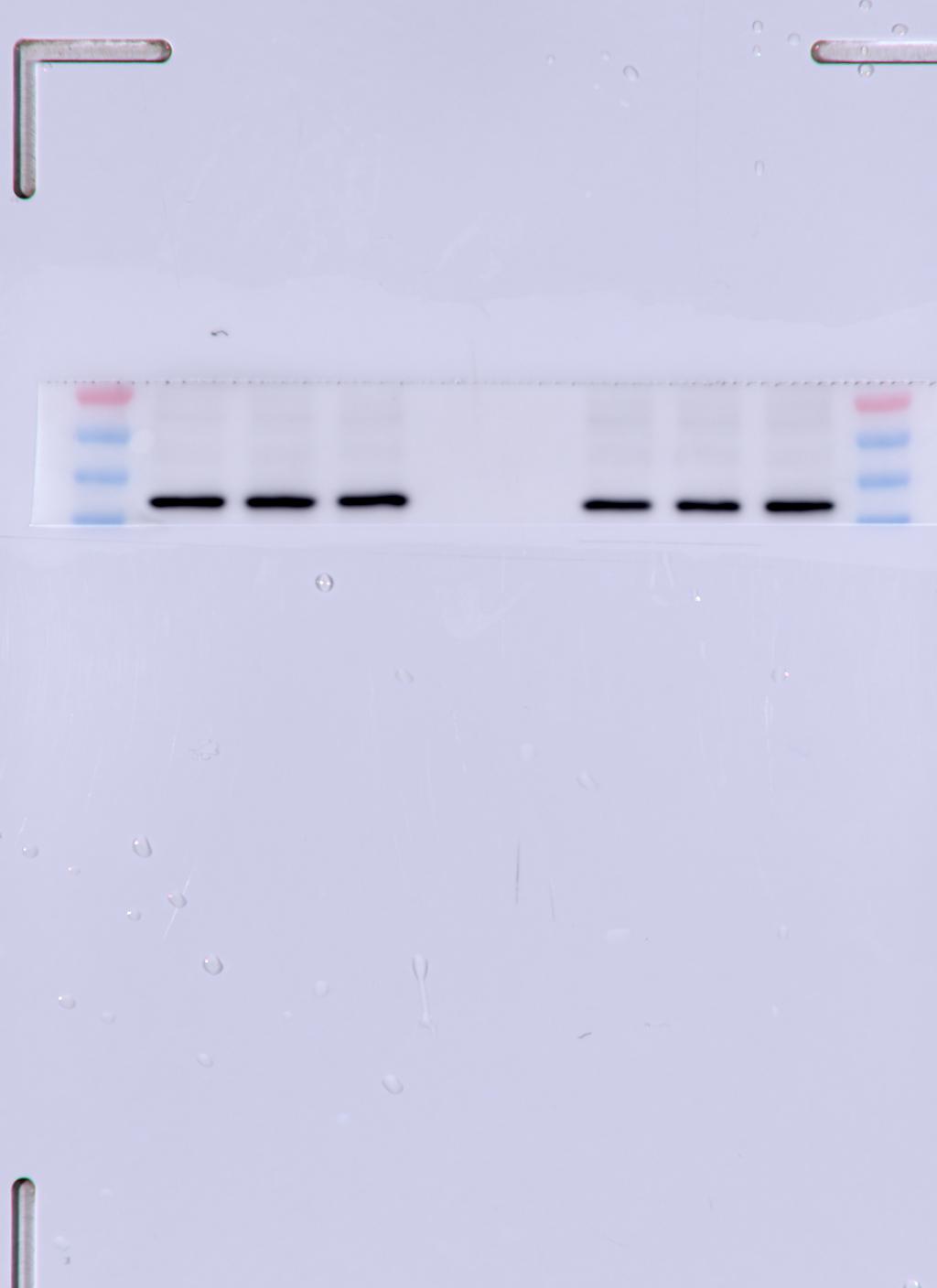

Supplement: Supplemental Information 5 [file peerj-09-12138-s005.zip › Fig 6D/GAPDH.jpg]

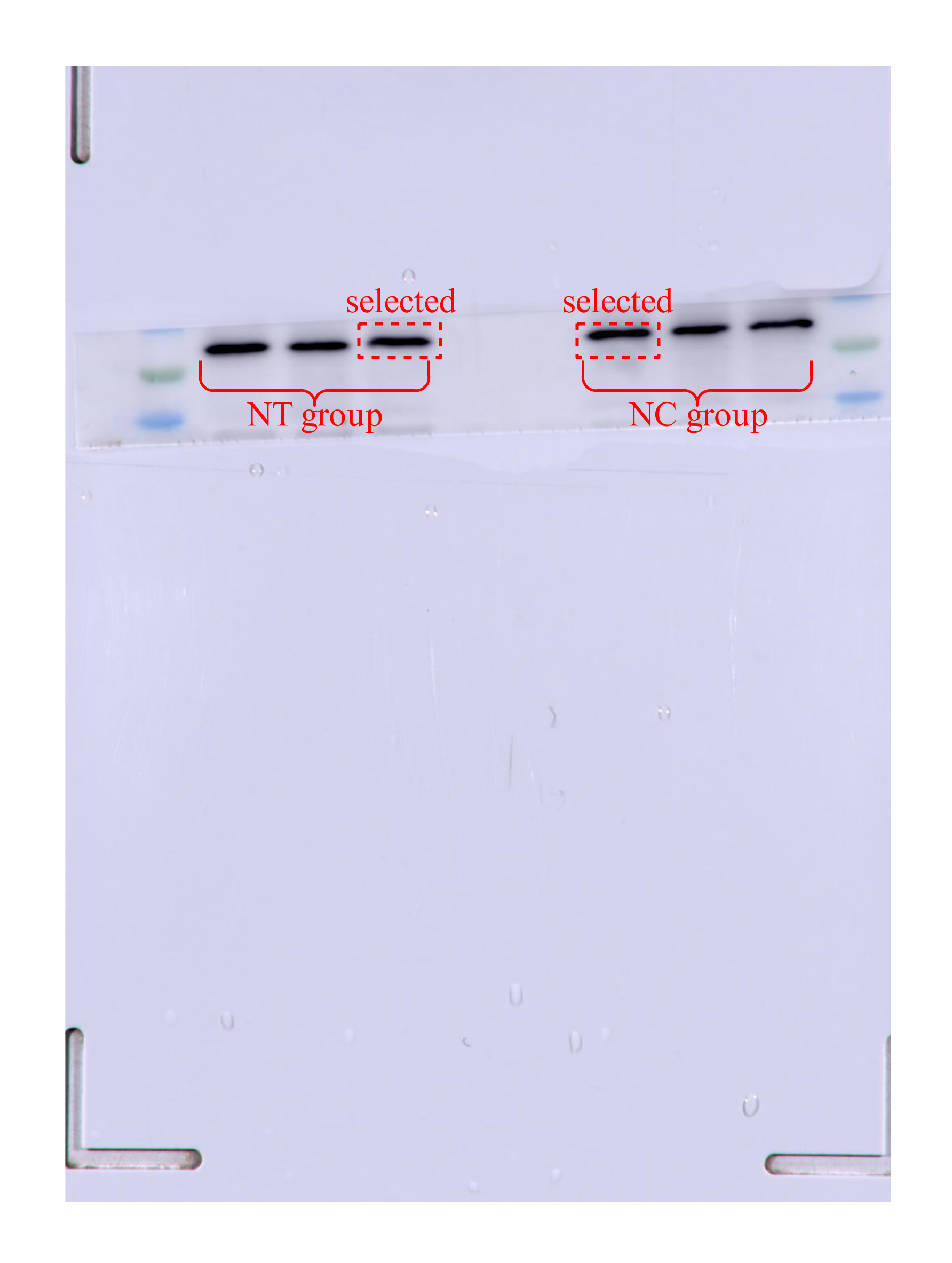

Supplement: Supplemental Information 5 [file peerj-09-12138-s005.zip › Fig 6D/mmp grouped.png]

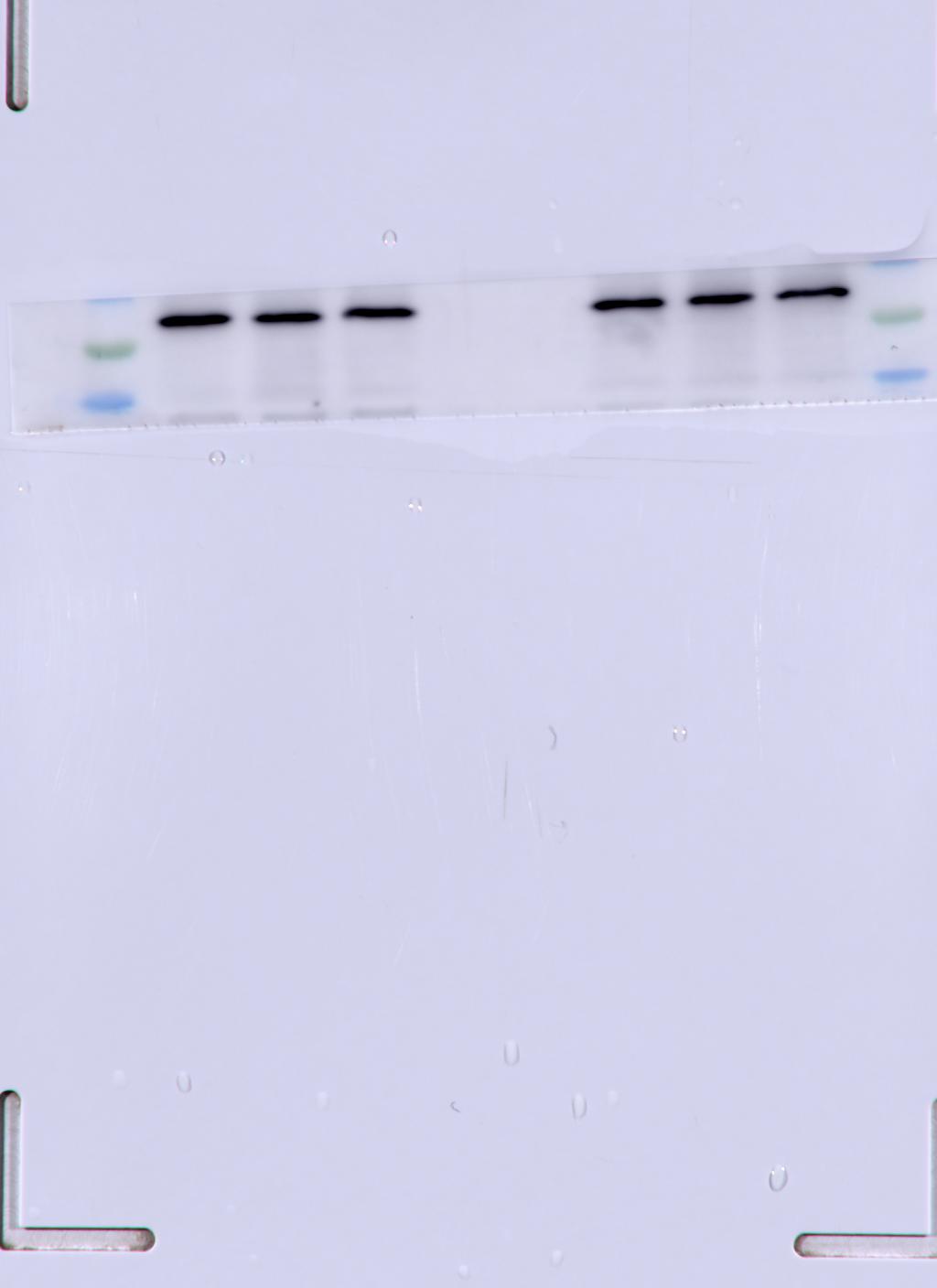

Supplement: Supplemental Information 5 [file peerj-09-12138-s005.zip › Fig 6D/mmp.jpg]

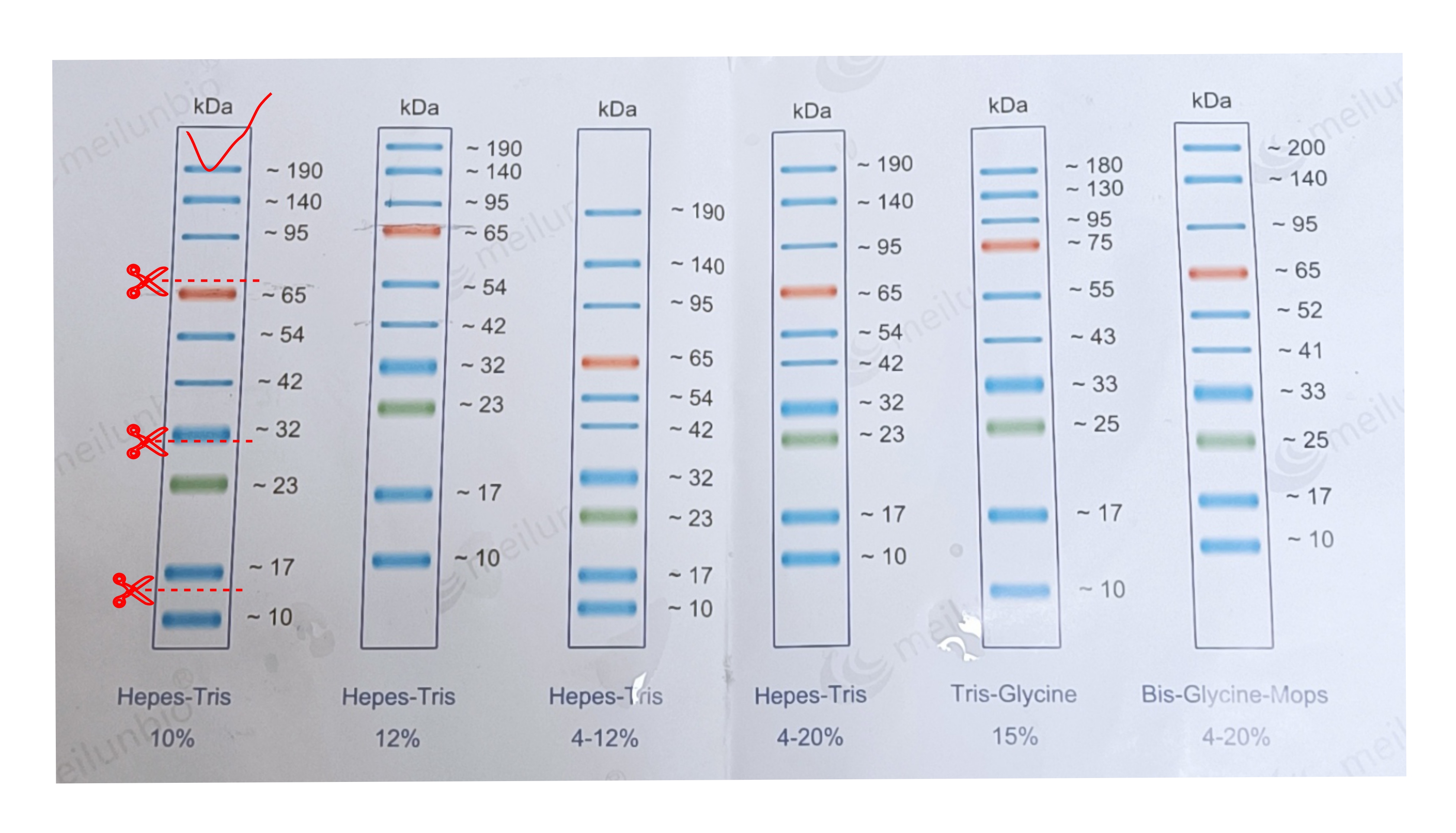

Supplement: Supplemental Information 5 [file peerj-09-12138-s005.zip › Fig 6D/protein marker.jpg]

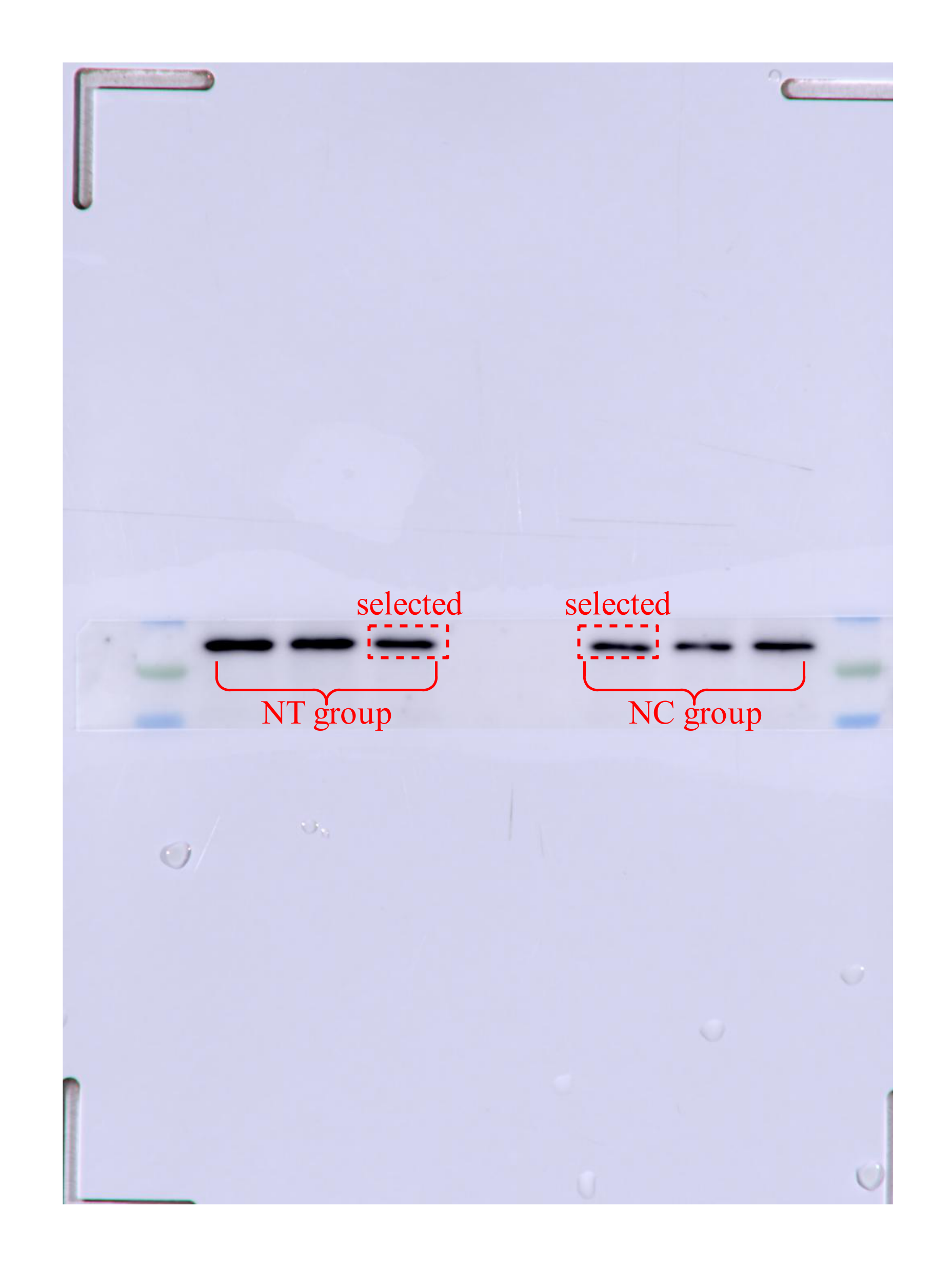

Supplement: Supplemental Information 5 [file peerj-09-12138-s005.zip › Fig 6D/timp grouped.png]

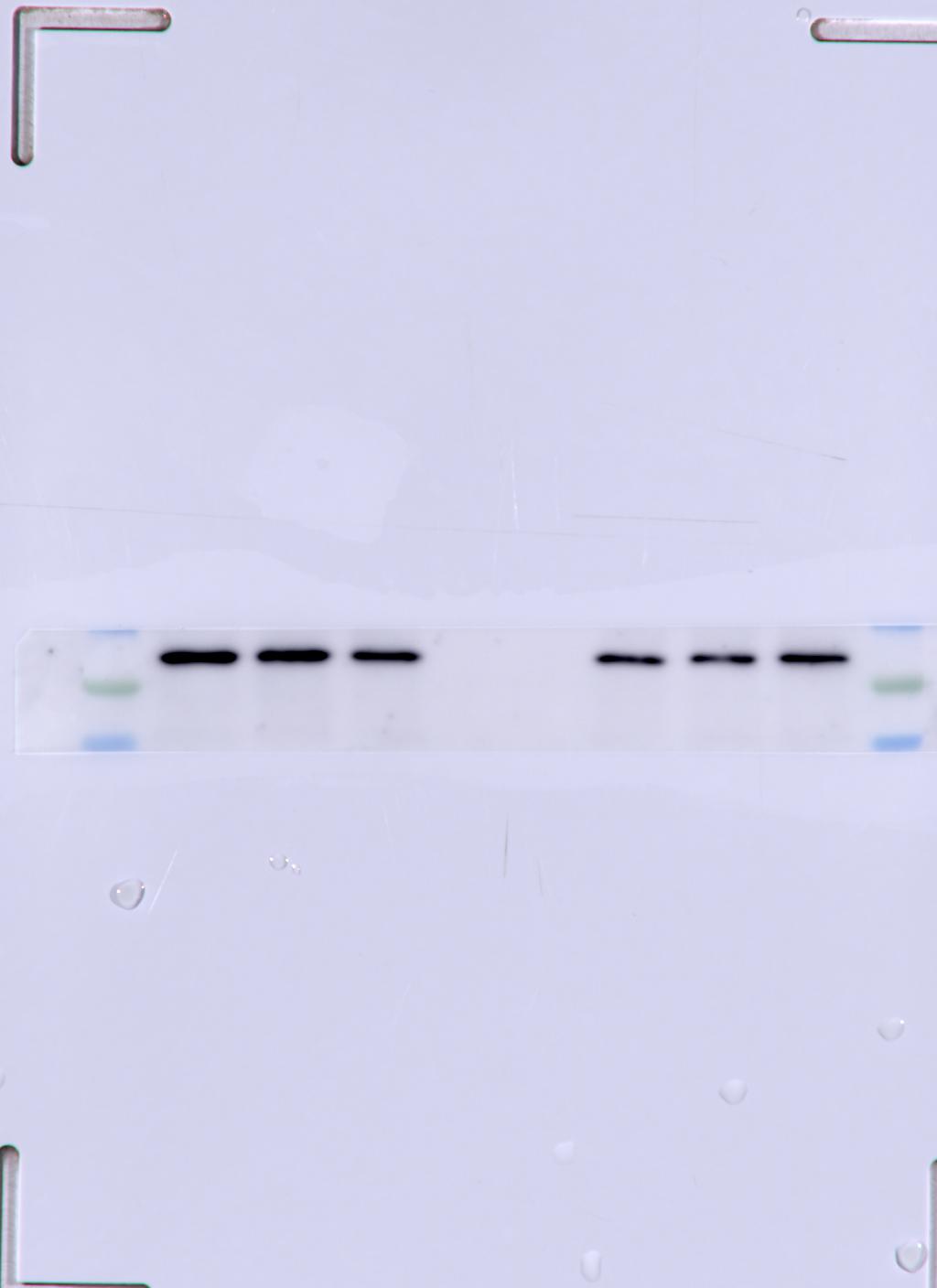

Supplement: Supplemental Information 5 [file peerj-09-12138-s005.zip › Fig 6D/timp.jpg]
